# Supplementary material for: Brain maps of general cognitive function and spatial correlations with neurobiological cortical profiles
Source: bioRxiv. 2024 Dec 18:2024.12.17.628670. Preprint. [Version 2] doi: 10.1101/2024.12.17.628670 (PMC11702631; doi:10.1101/2024.12.17.628670)
Supplement: Supplement 1 [file media-1.pdf]

Cognitive descriptives/models

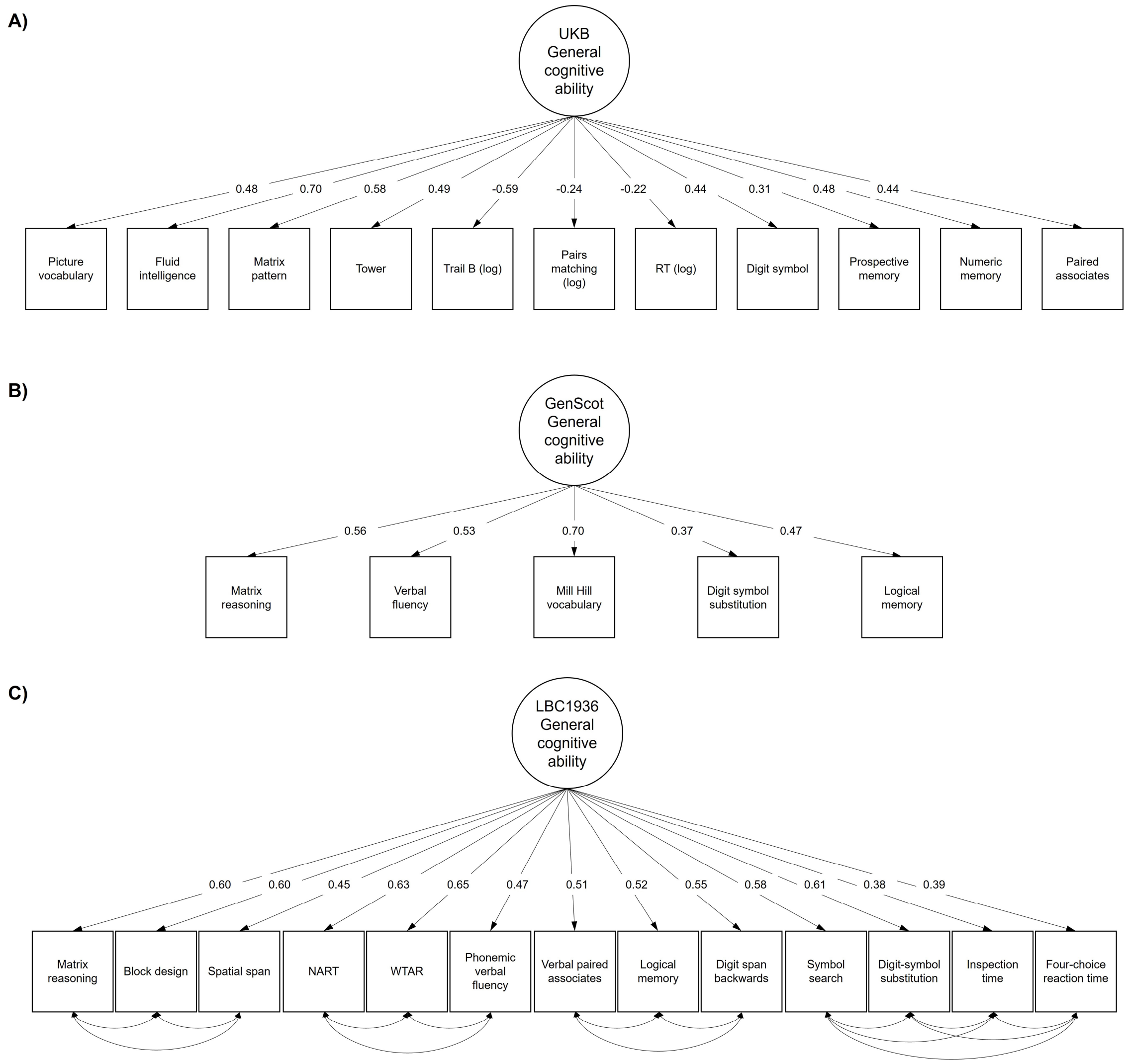

Figure S1 Simplified path diagrams of cognitive ability latent models alongside density plots of the final cognitive ability prediction *z* scores for A) UKB, B) GenScot, and C) LBC1936. The within-domain residual variances for LBC1936 are in Table S8.

Vertex-wise descriptive stats

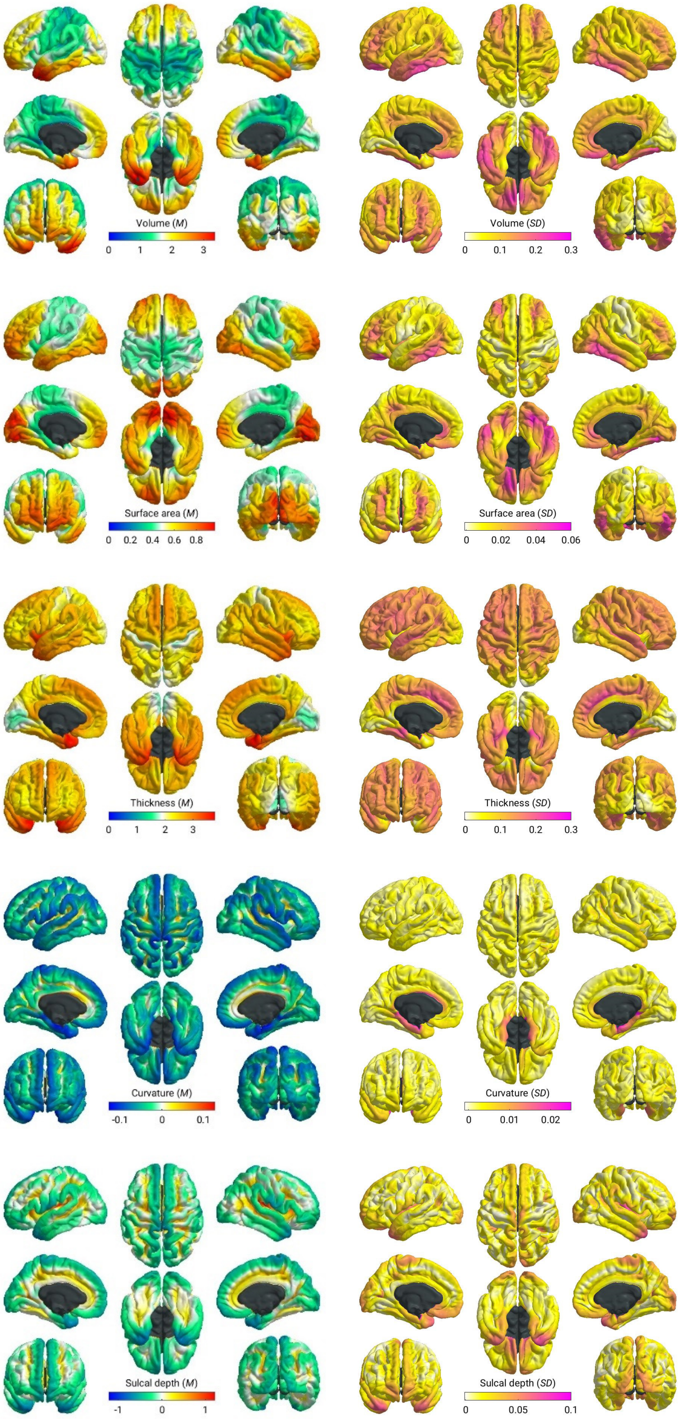

Figure S2 Meta-analysed mean (left) and *SD* (right) values for each cortical morphometry measure. Mean volume is in mm^3, surface area is in mm^2, thickness is in mm, curvature in in mm^{-1}, sulcal depth is in mm.

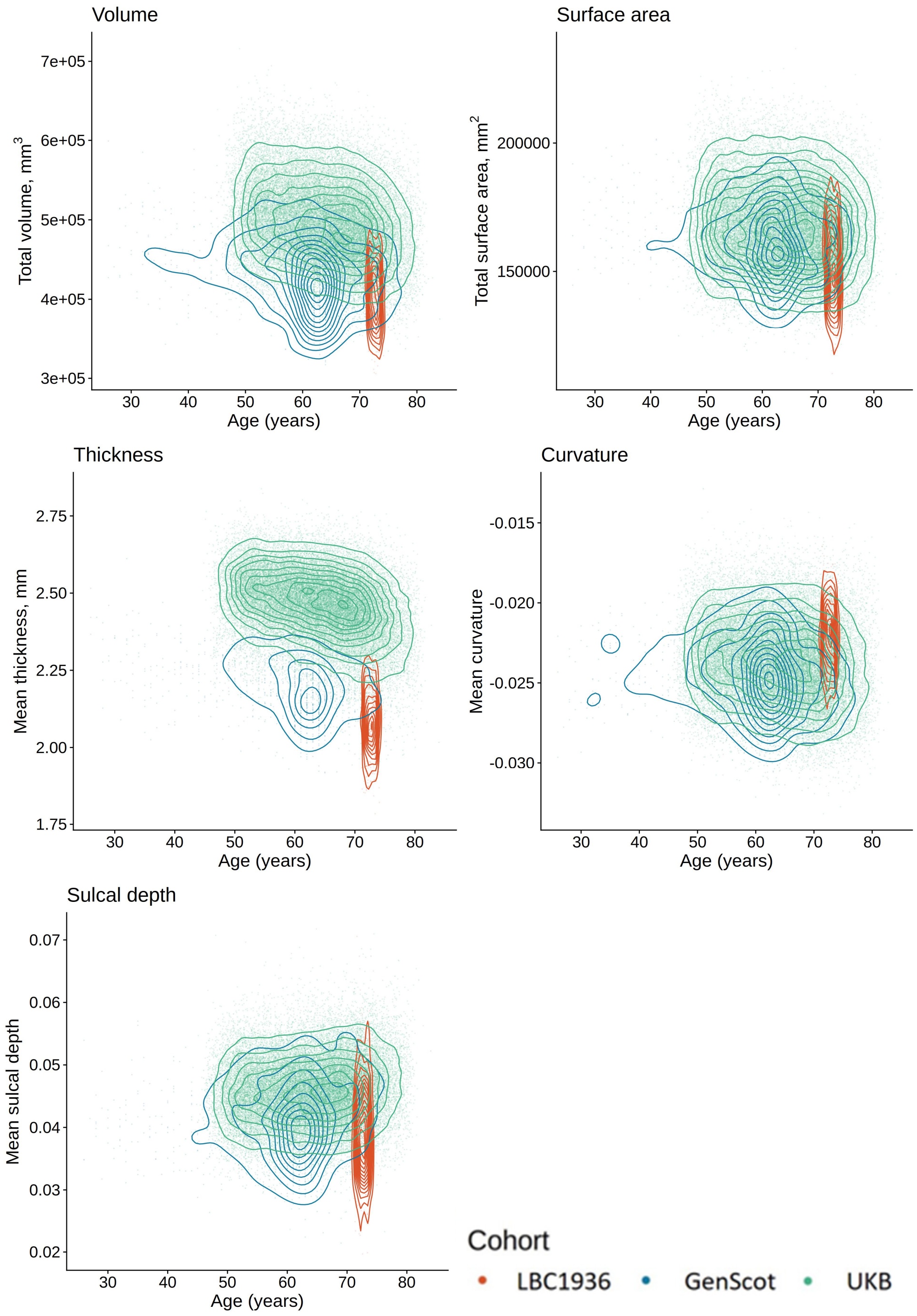

Figure S3 Raw participant totals/means of the 5 vertex-wise measures, plotted by age and cohort.

Smoothing tolerances

LBC1936

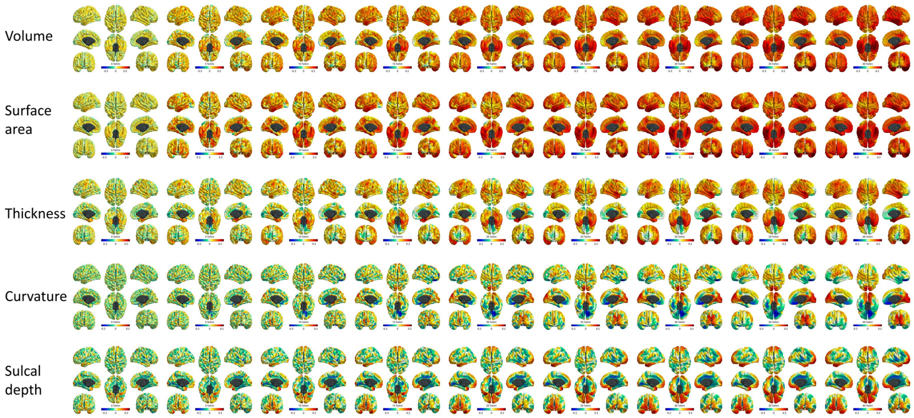

GenScot

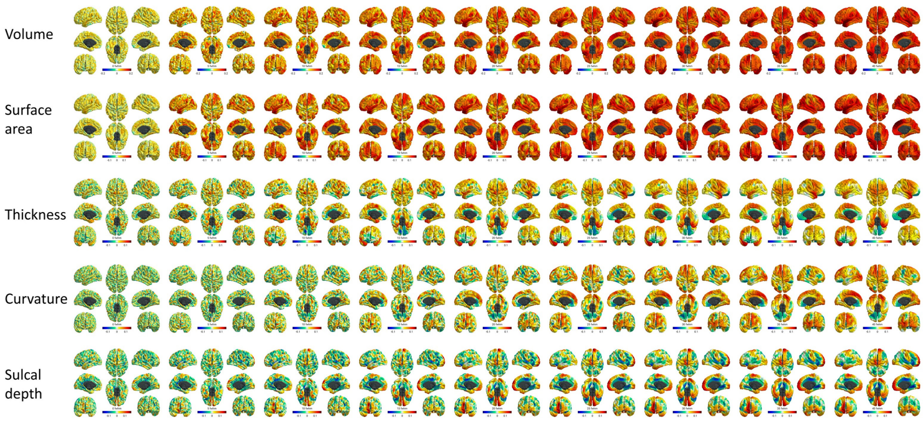

UKB

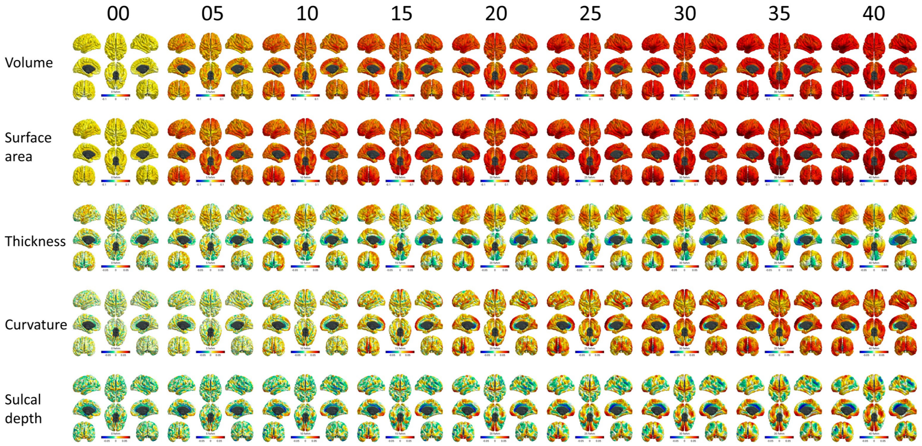

Figure S4 Maps of g-associations in each cohort at the 9 smoothing tolerances for the 5 morphometry measures.

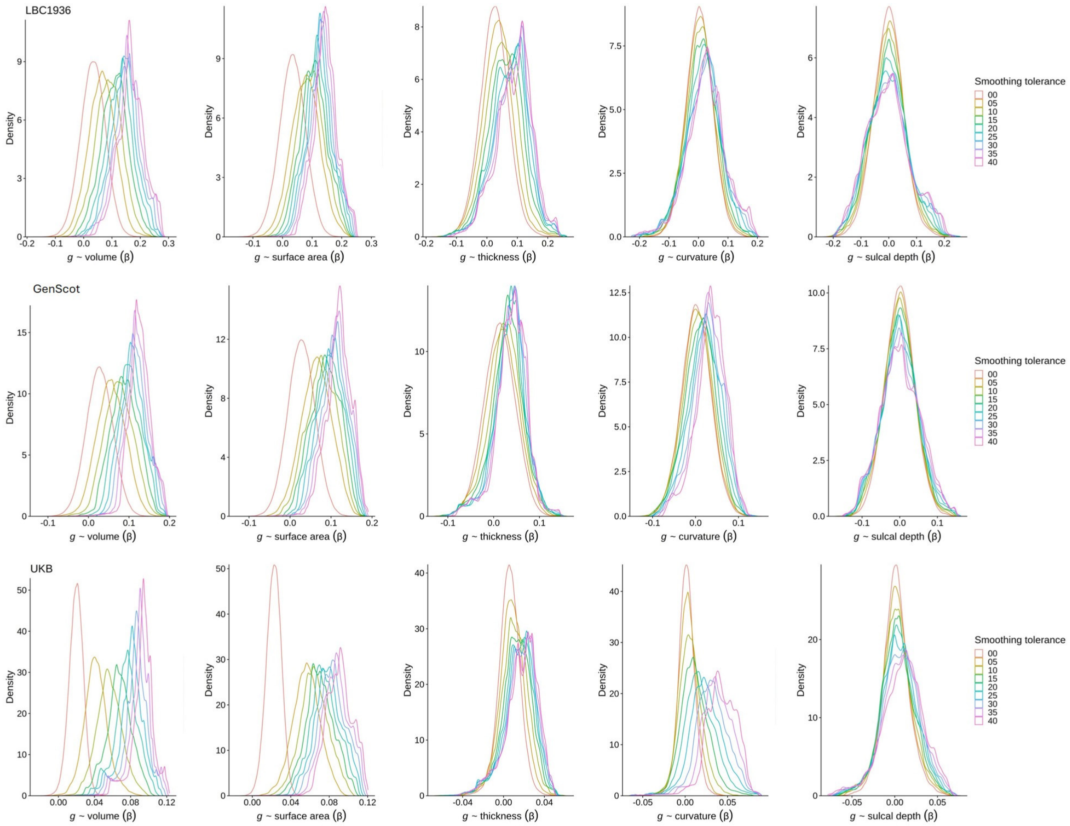

Figure S5 Density plots showing *g*-associations for each cohort for each of the 9 smoothing tolerances (0, 5, 10, 15, 20, 25, 30, 35 and 40 mm FWHM).

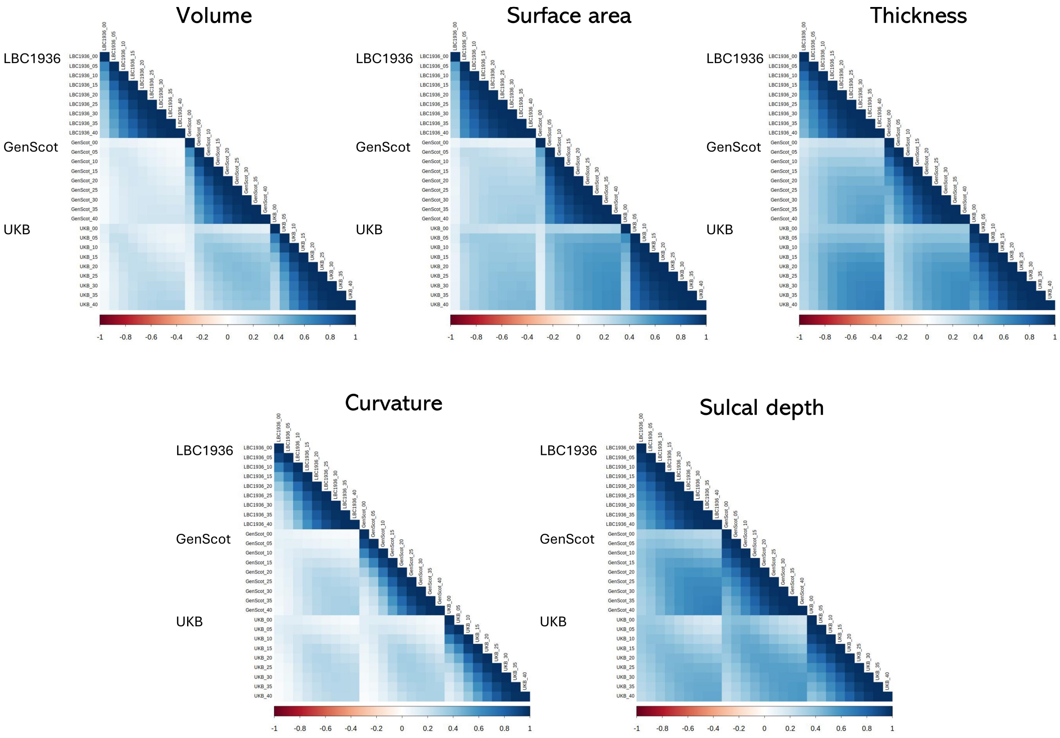

Figure S6 Within and between cohort spatial correlations of vertex-wise *g*-associations for the 5 cortical morphometry measures at each of the 9 smoothing tolerances (0, 5, 10, 15, 20, 25, 30, 35, 40 mm FWHM). Between-cohort spatial correlations increase with increasing smoothing tolerance, as data converges towards the total surface estimates, while within-cohort correlations decrease with increasing smoothing tolerances.

Vertex-wise meta-analysis results

g associations

_g_ ~ volume

LBC1936 GenScot UKB

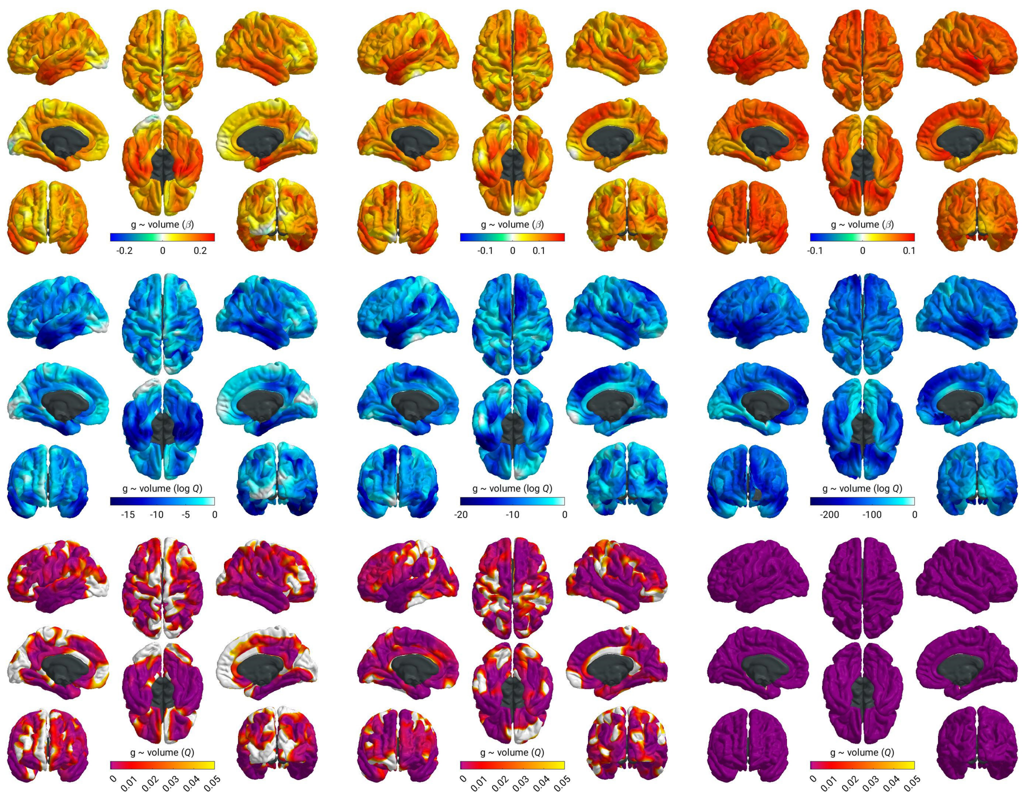

Figure S7 *g*-volume associations for each cohort at 20 FWHM (top = β, middle = log *FDR Q*, bottom = *FDR Q*). The beta and log *FDR Q* scales are set at the maximum values for the relevant cohort across all measures.

***g* ~ surface area**

LBC1936 GenScot UKB

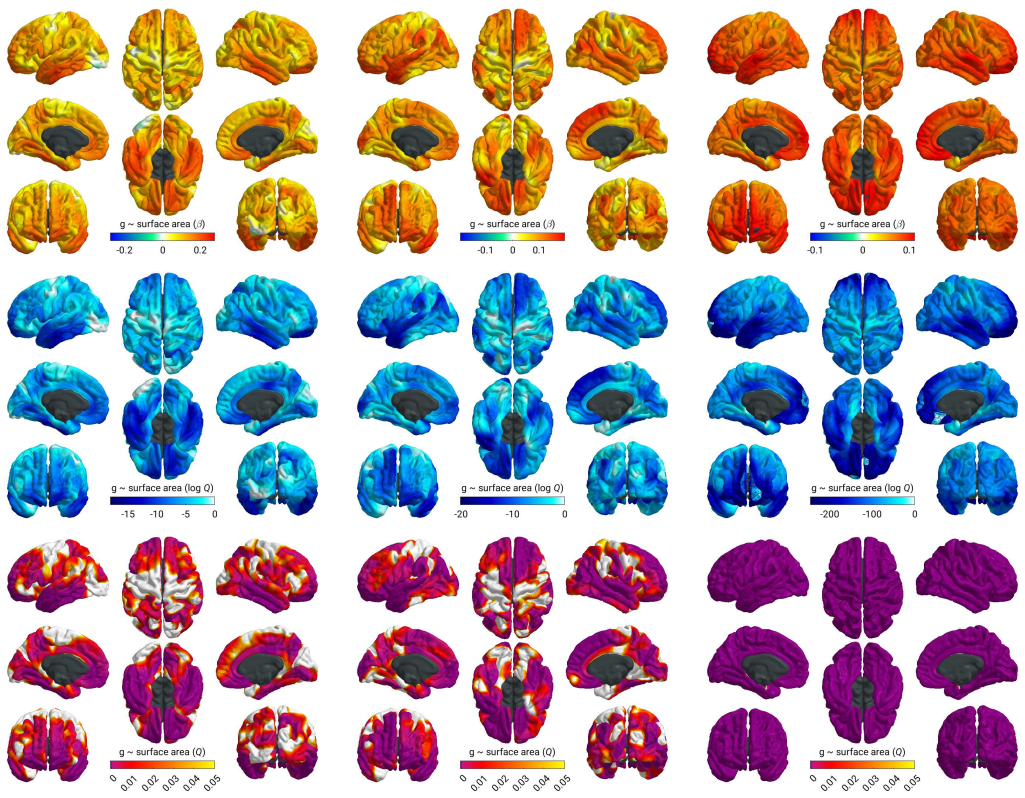

Figure S8 g-surface area associations for each cohort at 20 FWHM (top = β, middle = log *FDR Q*, bottom = *FDR Q*). The beta and log *FDR Q* scales are set at the maximum values for the relevant cohort across all measures.

g ~ thickness

LBC1936 GenScot UKB

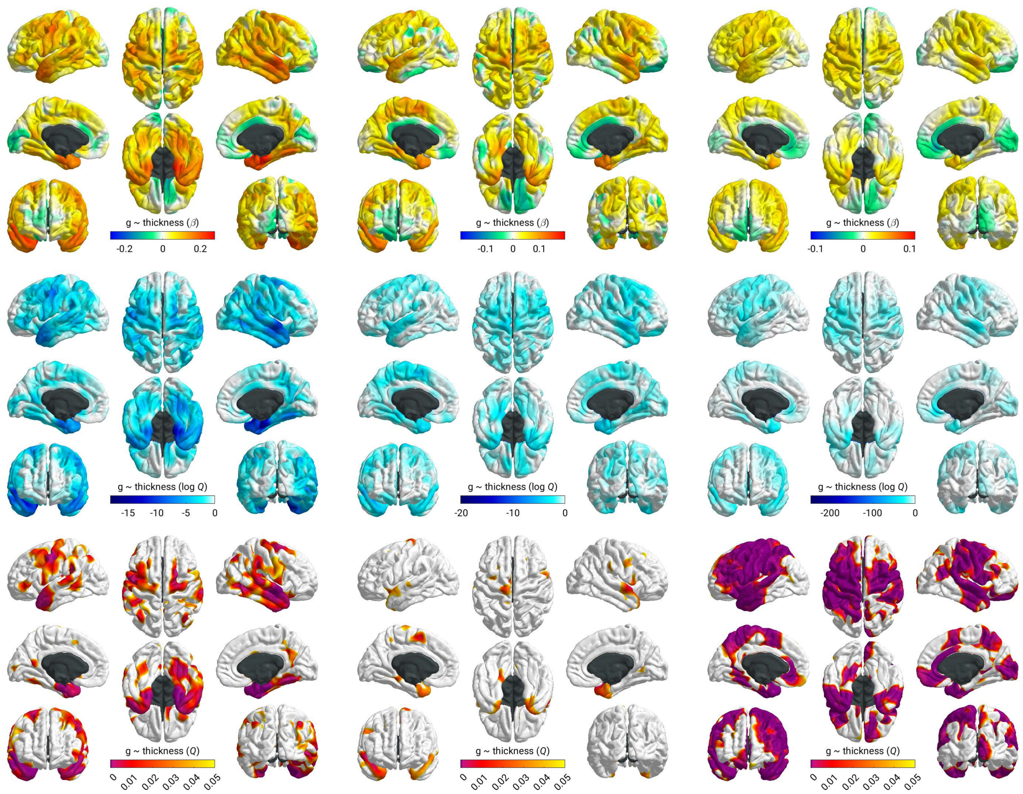

Figure S9 g-thickness associations for each cohort at 20 FWHM (top = β, middle = log *FDR Q*, bottom = *FDR Q*). The beta and log *FDR Q* scales are set at the maximum values for the relevant cohort across all measures.

g ~ curvature

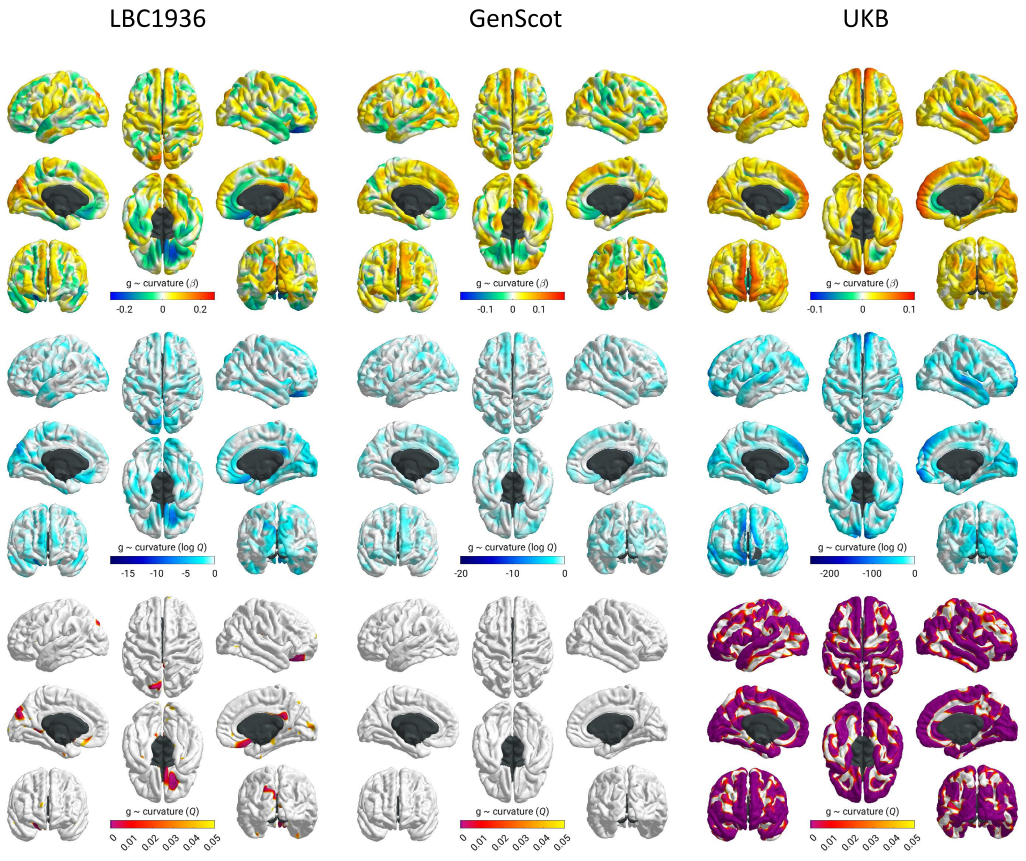

Figure S10 g-curvature associations for each cohort at 20 FWHM (top = β, middle = log *FDR Q*, bottom = *FDR Q*). The beta and log *FDR Q* scales are set at the maximum values for the relevant cohort across all measures.

g ~ sulcal depth

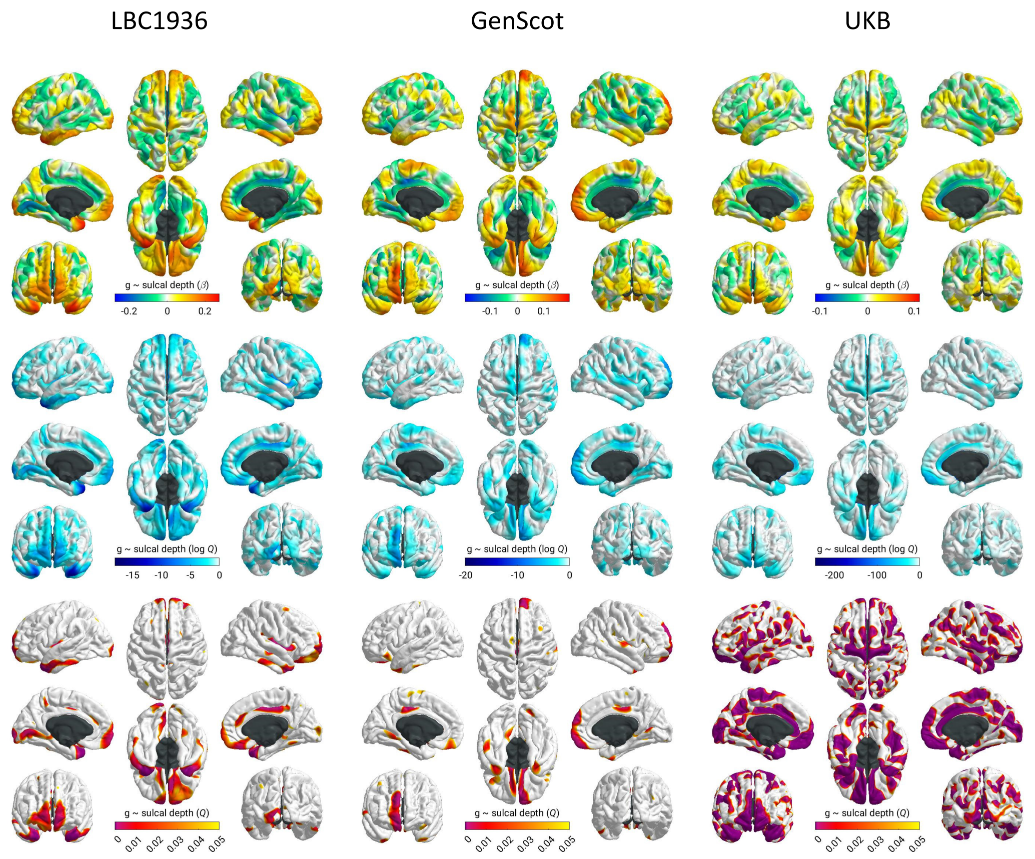

Figure S11 *g*-sulcal depth associations for each cohort at 20 FWHM (top = β, middle = log *FDR Q*, bottom = *FDR Q*). The beta and log *FDR Q* scales are set at the maximum values for the relevant cohort across all measures.

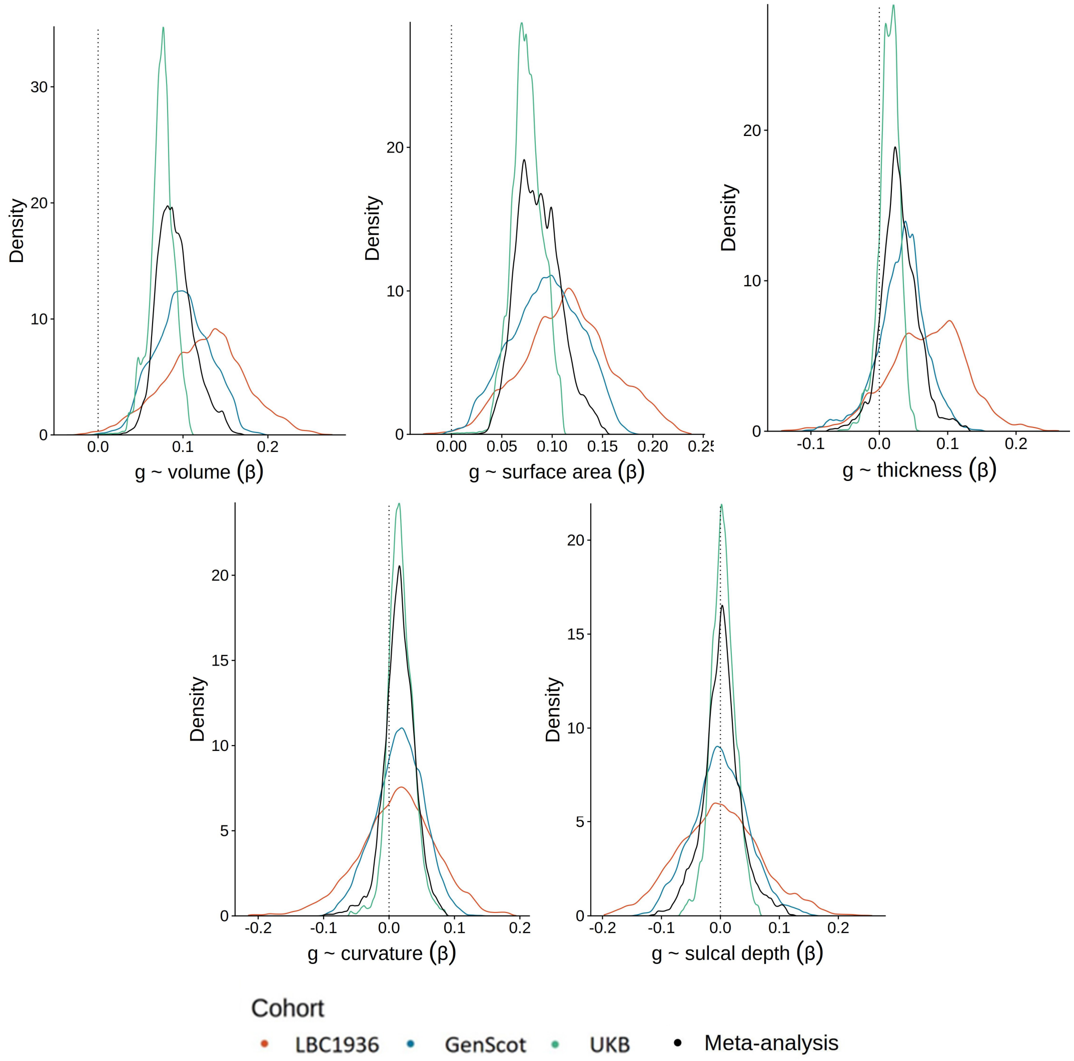

Figure S12 Density distributions of *g*-association β values for the three cohorts and the meta-analysed *g*-associations for the 5 vertex-wise measures of morphometry. The vertical dotted line marks β = 0.

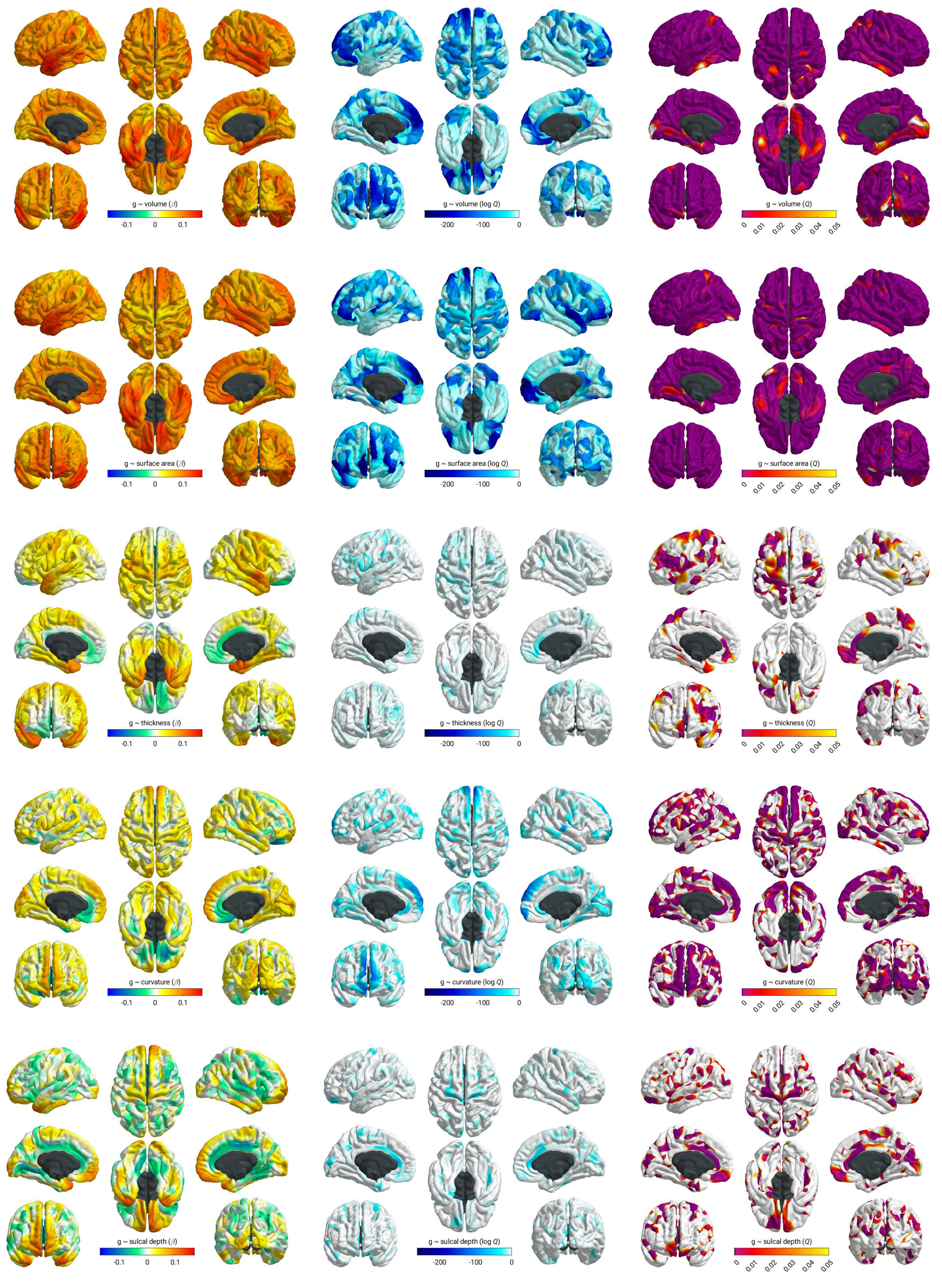

Figure S13 Meta-analysed β, log *FDR Q* and *FDR Q* values for *g* ~ morphometry associations for the 5 vertex-wise measures (from top to bottom: volume, surface area, thickness, curvature and sulcal depth). The scale limits for the β maps set at 0.17+/-, which is the maximum absolute value for any measure. The scale limits for log *Q* maps is set at the minimum value for any measure (which is -263.24, or *FDR Q* = 4.75×10^{-115}).

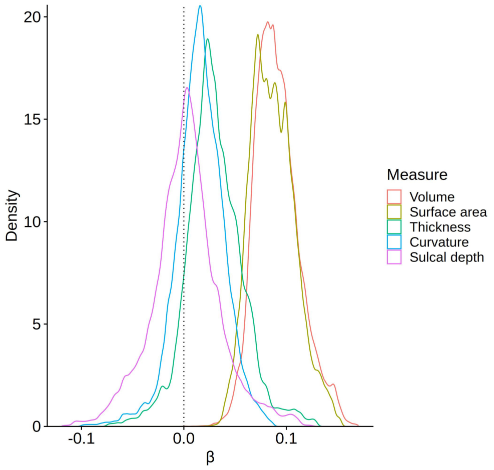

Figure S14 Density distributions for the meta-analysed *g* ~ morphometry associations for the 5 measures of morphometry (volume, surface area, thickness curvature and sulcal depth). The vertical dotted line marks β = 0.

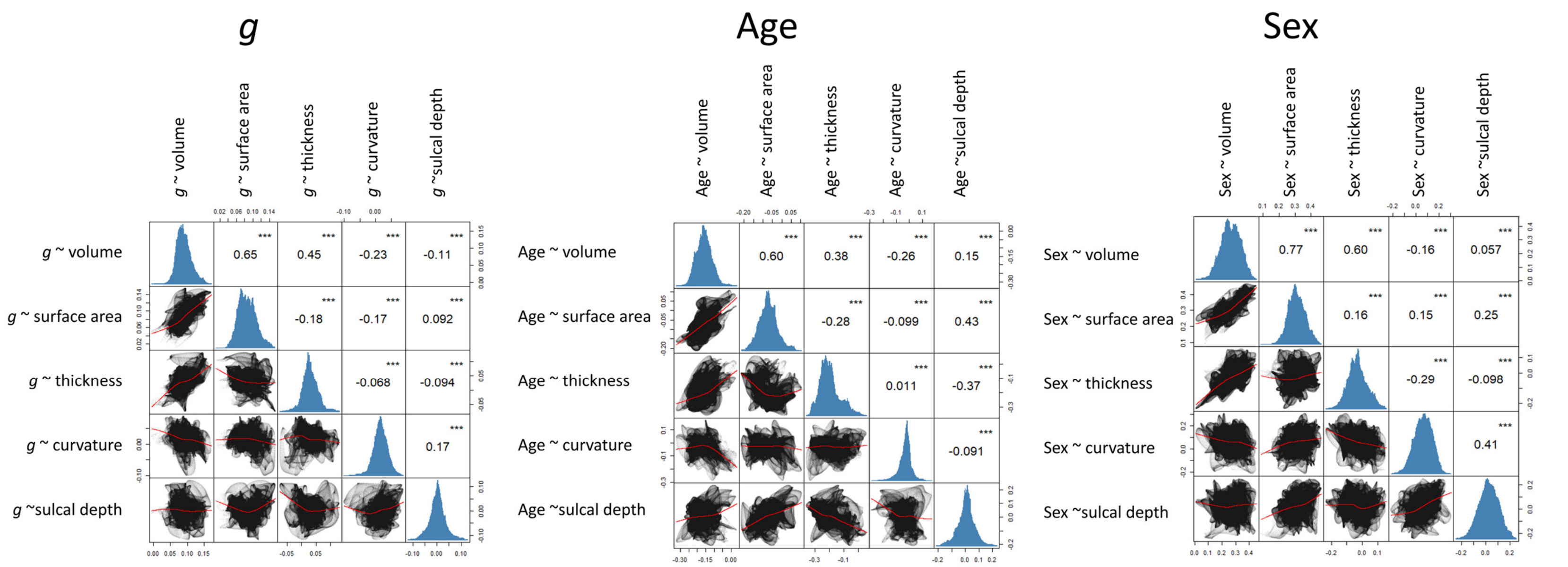

Figure S15 Spatial correlation plots of the meta-analysed β values for vertex-wise associations of 5 measures of morphometry (volume, surface area, thickness, curvature and sulcal depth) and (left) *g*, (middle) age and (right) sex. These are linked to Table 3 in the main text.

Metabolism

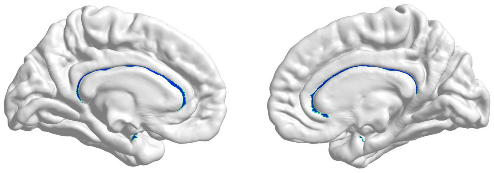

Figure S16 The metabolism data was registered from fsLR 164k to fsaverage 164k, and there was a slight difference in the cortical masks, in that there were 2153 additional vertices included in the mask compared to the fsaverage mask. These vertices are shown in blue in this figure. They were not included in any of the correlations that include metabolism.

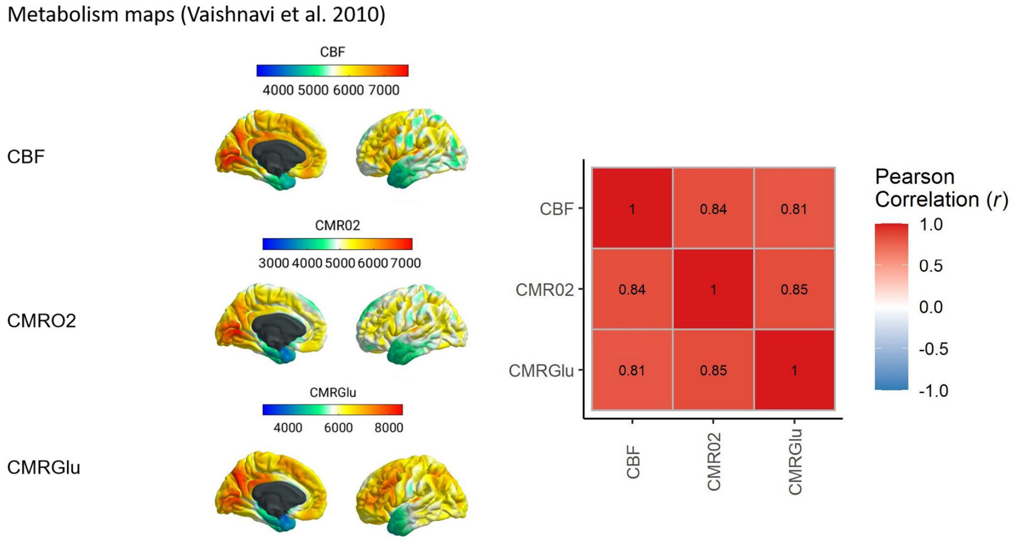

Figure S17 Left: Metabolism data mapped to the cortex. Right: Spatial correlation plot showing the high correlations between three measures of cortical metabolism, which justifies a principal component analysis to create one measure of cortical metabolism.

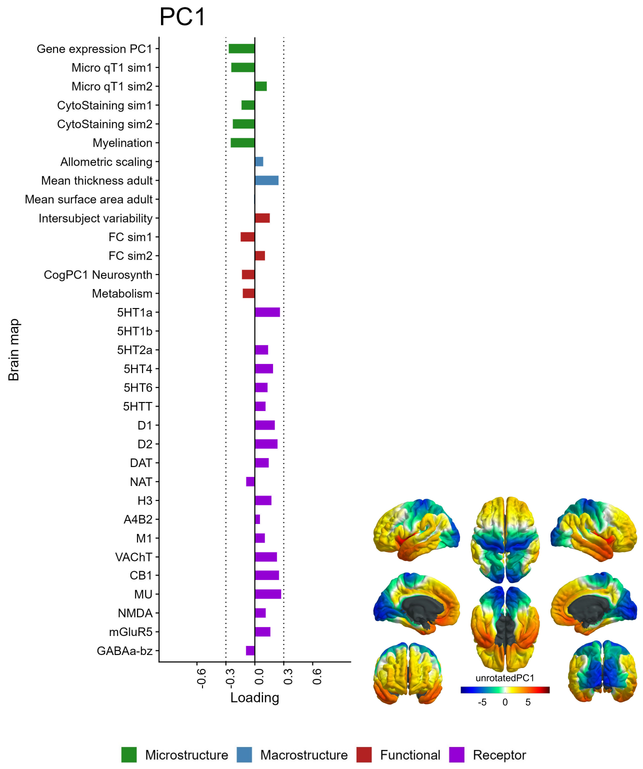

Figure S18 Unrotated PC1 loadings. Note, the coefficient of factor congruence between the unrotated PC1 and PC1 after varimax rotation with 4 components was 0.9.

Global and subcortical brain structure descriptives

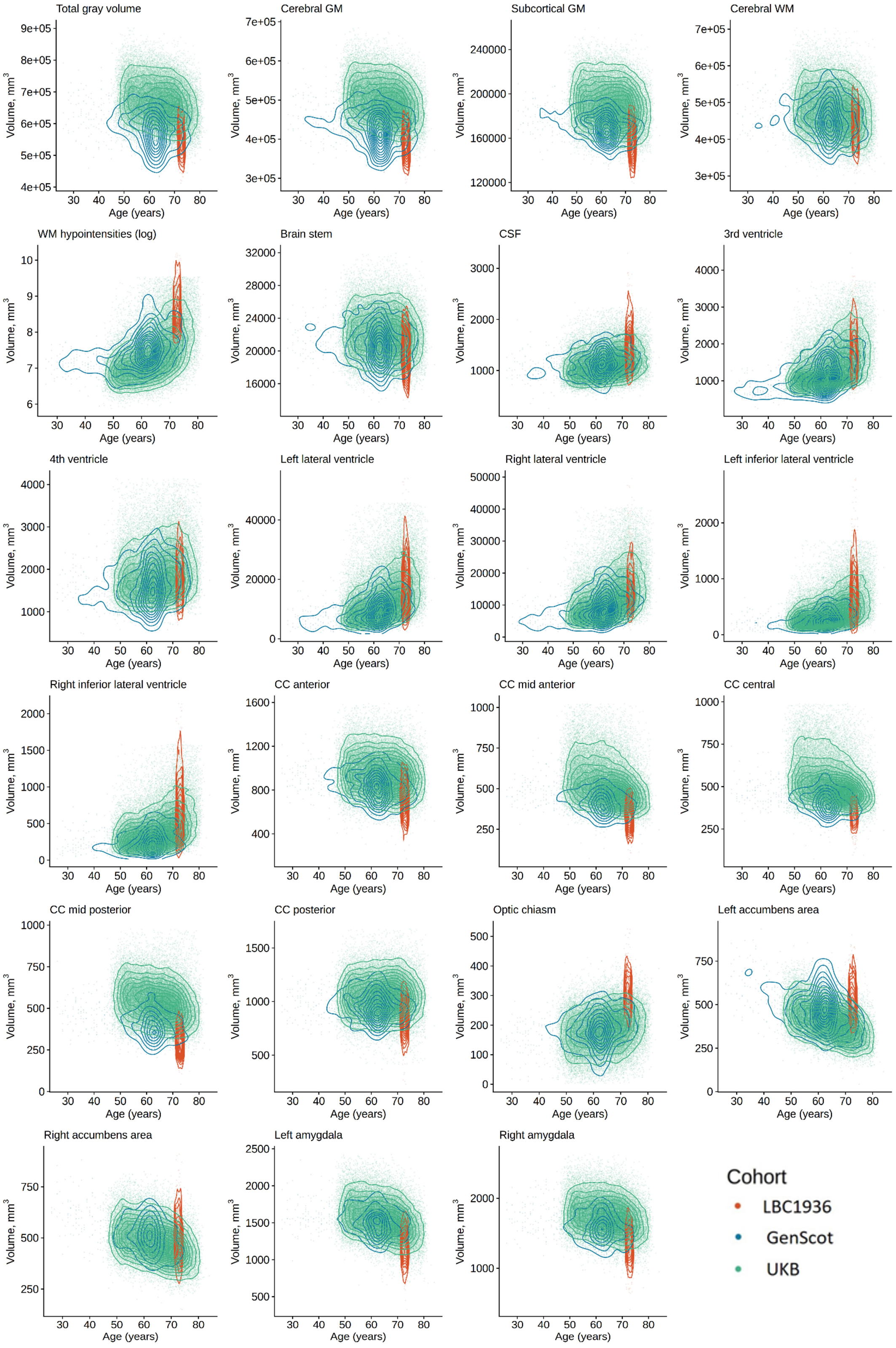

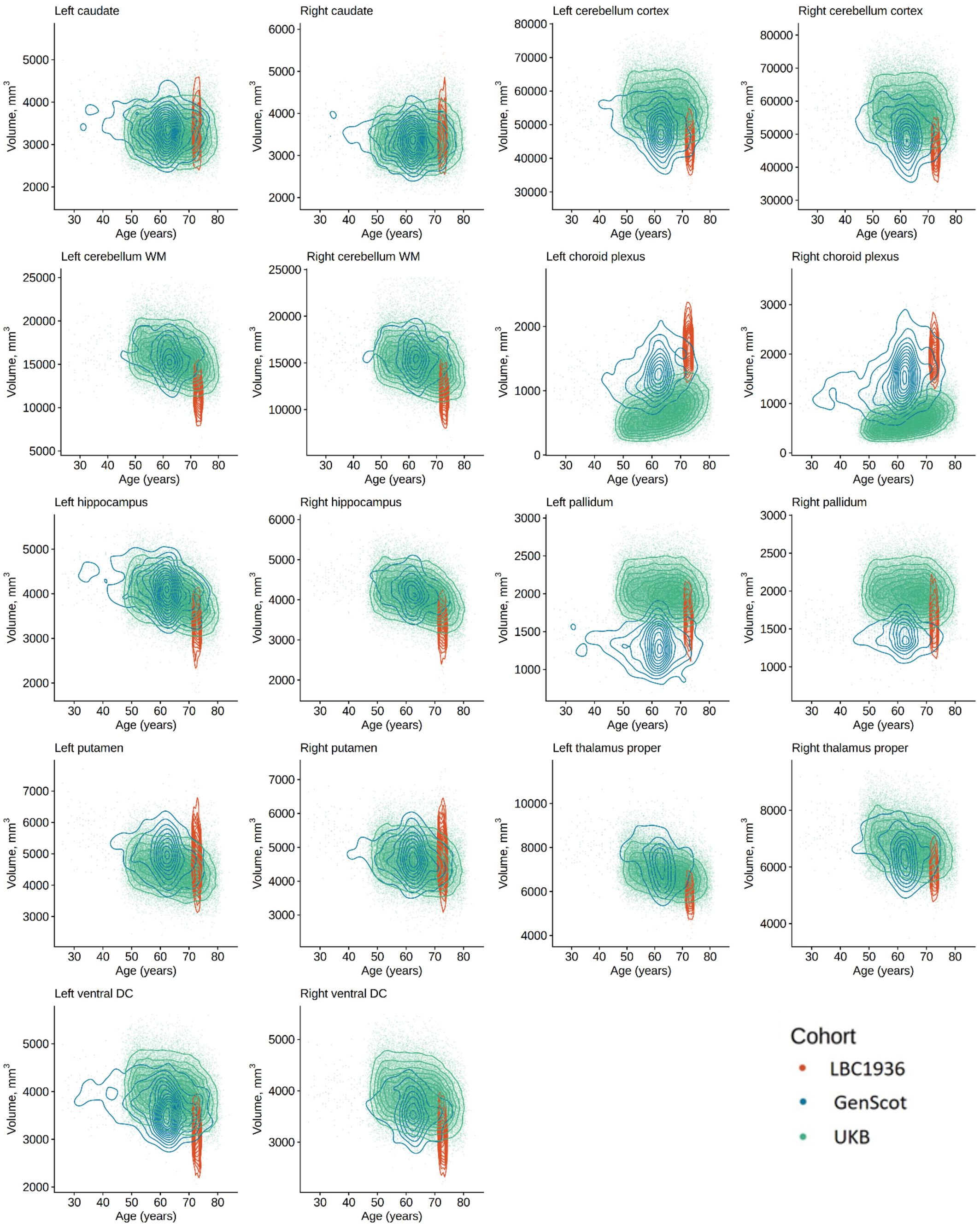

Figure S19 Raw data plots of the volume of each global and subcortical brain structure, coloured by cohort.

Neurobiological profiles

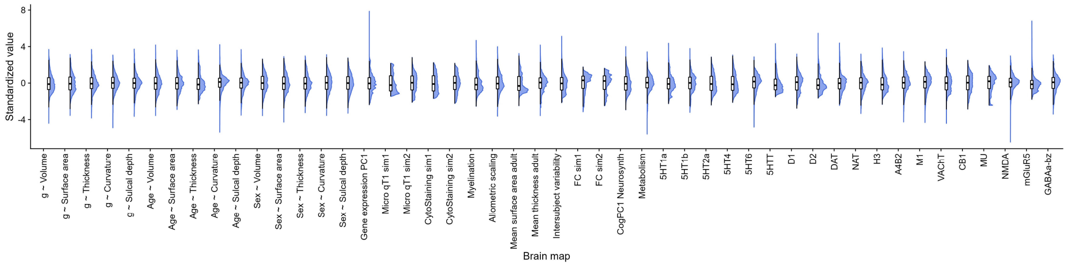

Figure S20 Density distributions of all the vertex-wise maps (g-associations, age-associations, sex-associations and 33 neurobiological profiles)

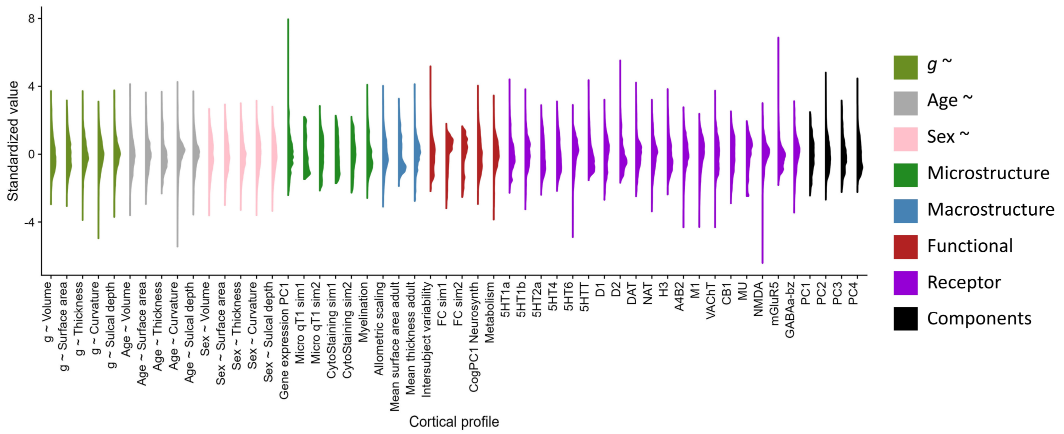

Figure S21 Distributions of standardized values for all cortical profiles, including PCs, coloured by profile type.

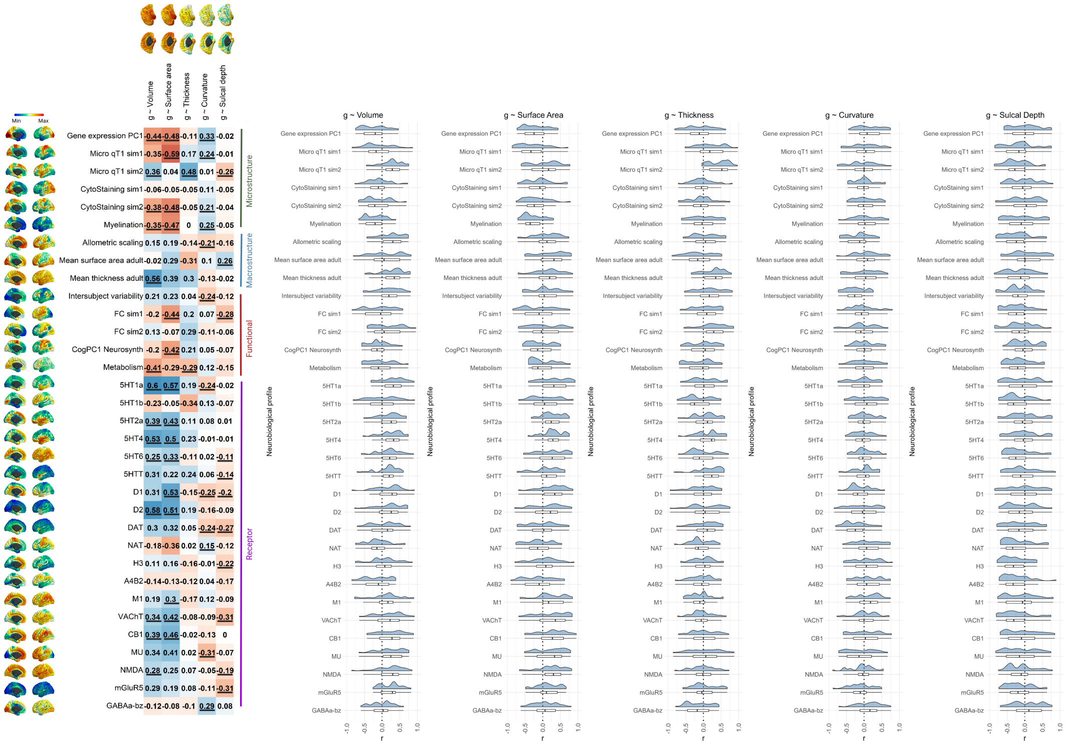

Figure S22 Extended version of main text Figure 5. A) Correlation plots showing spatial correlations between g-vertex-wise morphometry associations and various multiscale cortical profiles Those with *p_spin* values < .05 are underlined. B) Summary data of regional correlations for the cortex-level correlations in A).

Regional correlations

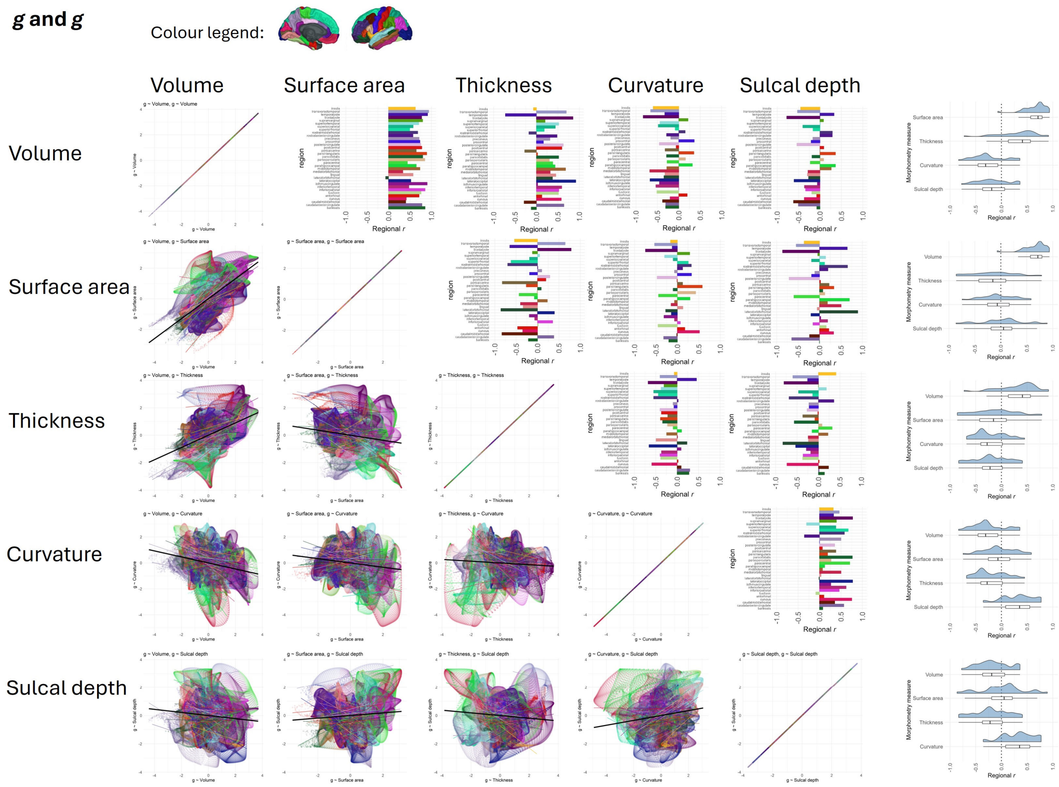

Figure S23 Within-region vertex-wise spatial correlations for **different morphometry measures with *g***. A) scatterplots showing each correlation, coloured by Desikan-Killiany region, B) summary of regional correlations, C) bar graph showing each regional correlation. The data underpinning these figures are in the Supplementary Tabular Data File.

Age and age

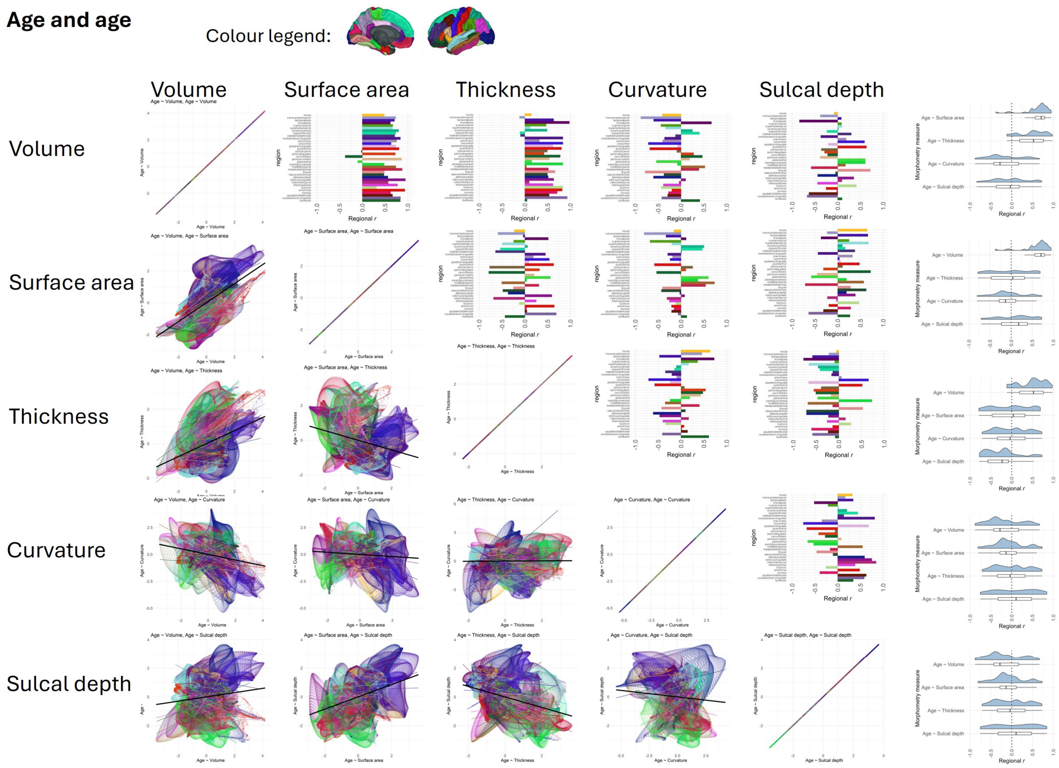

Figure S24 Within-region vertex-wise spatial correlations for **different morphometry measures with age.** A) scatterplots showing each correlation, coloured by Desikan-Killiany region, B) summary of regional correlations, C) bar graph showing each regional correlation. The data underpinning these figures are in the Supplementary Tabular Data File.

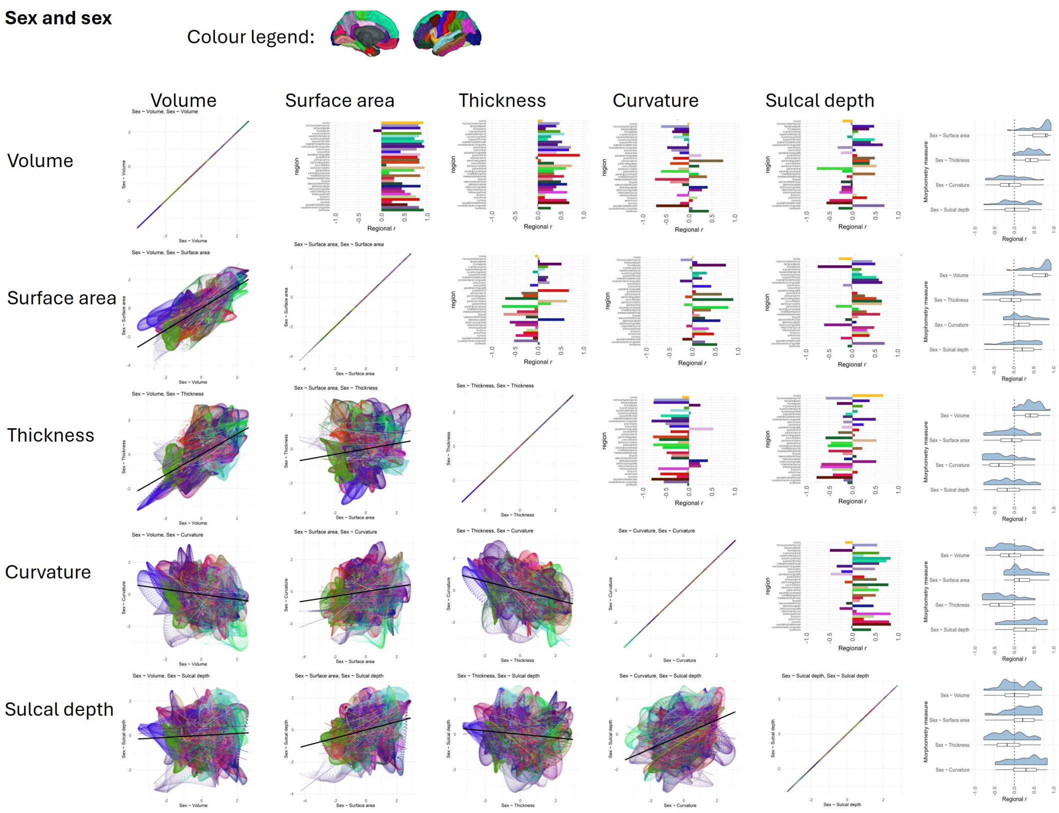

Figure S25 Within-region vertex-wise spatial correlations for **different morphometry measures with sex**. A) scatterplots showing each correlation, coloured by Desikan-Killiany region, B) summary of regional correlations, C) bar graph showing each regional correlation. The data underpinning these figures are in the Supplementary Tabular Data File.

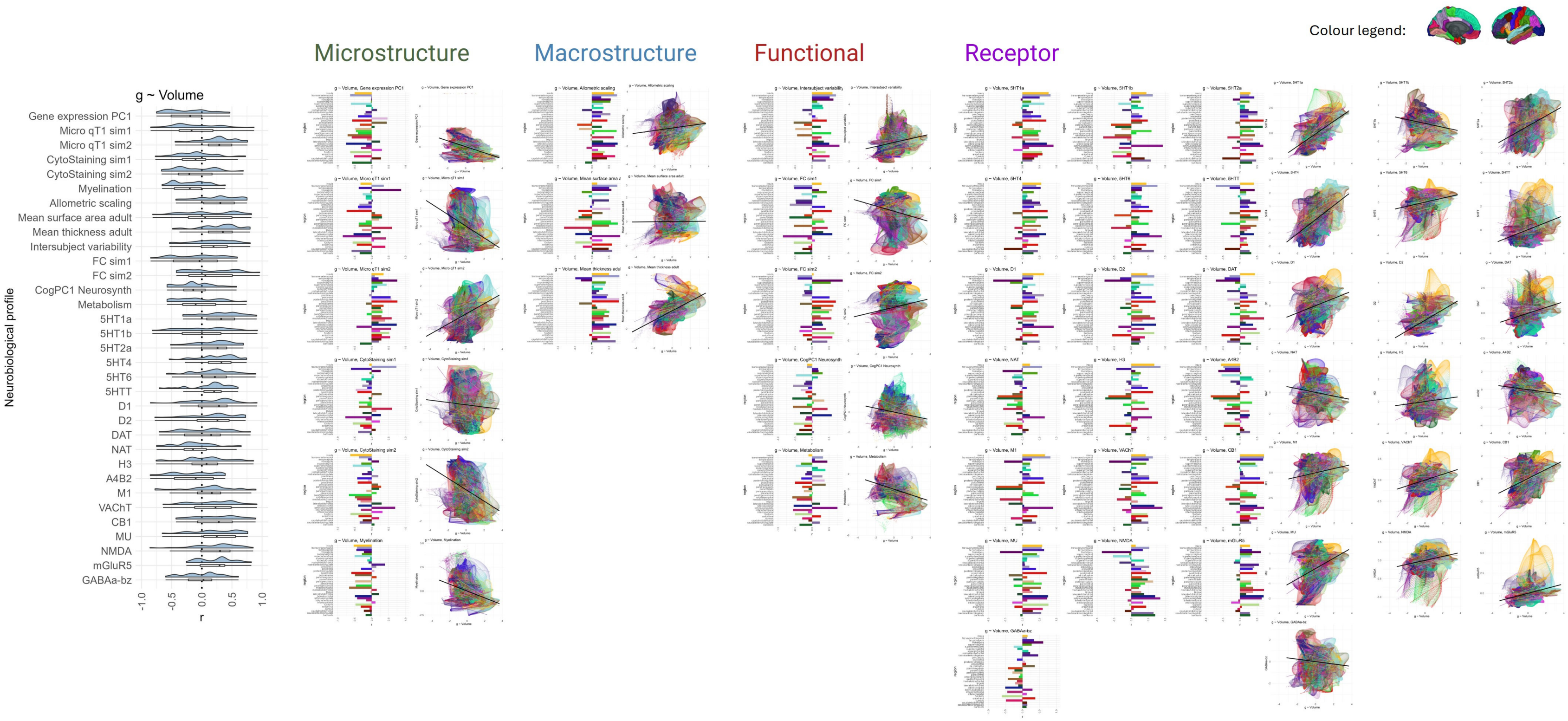

Figure S26 Within-region vertex-wise spatial correlations for ***g*-volume and neurobiological profiles** correlations. A) summary of regional correlations, B) bar graph showing each regional correlation and scatterplots showing each correlation, coloured by Desikan-Killiany region. The data underpinning these figures are in the Supplementary Tabular Data File.

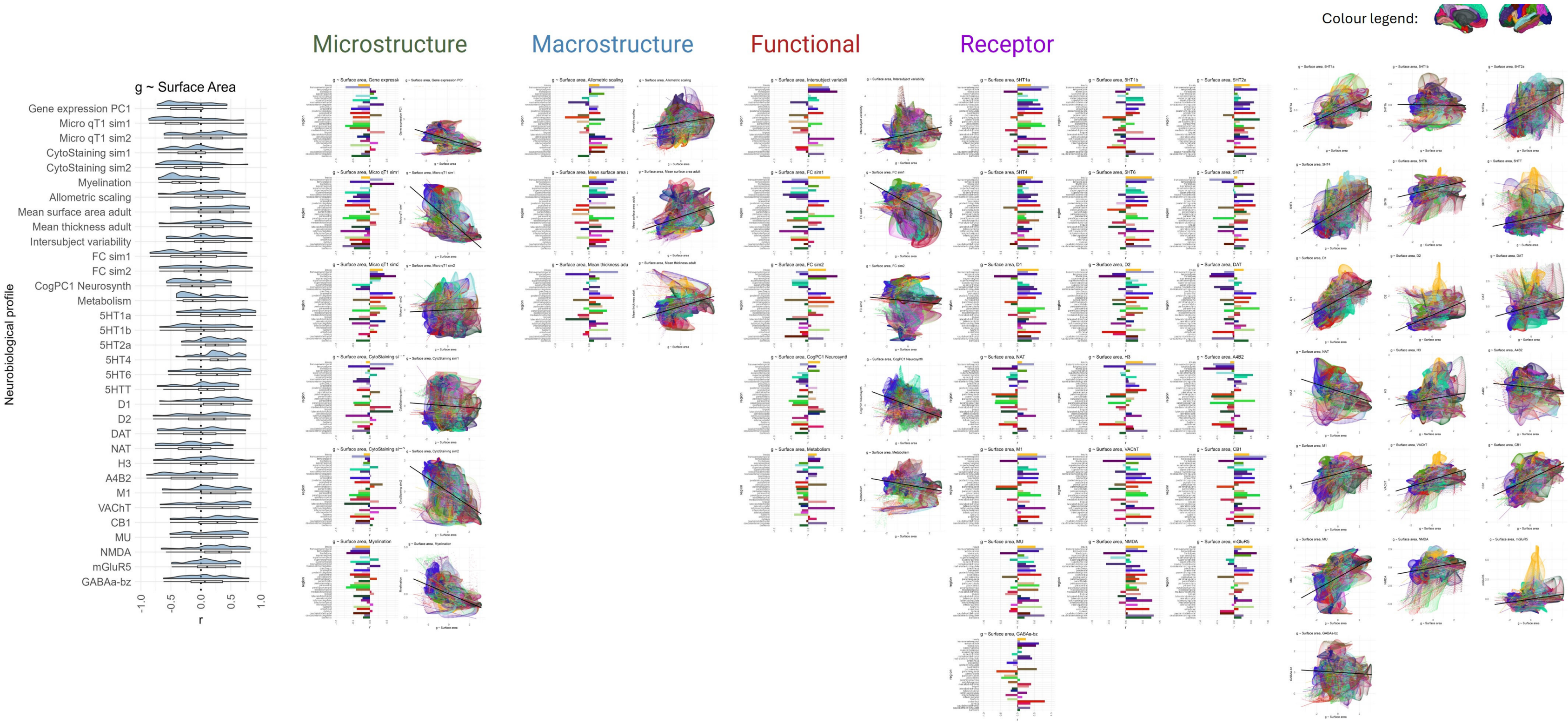

Figure S27 Within-region vertex-wise spatial correlations for ***g*-surface area and neurobiological profiles** correlations. A) summary of regional correlations, B) bar graph showing each regional correlation and scatterplots showing each correlation, coloured by Desikan-Killiany region. The data underpinning these figures are in the Supplementary Tabular Data File.

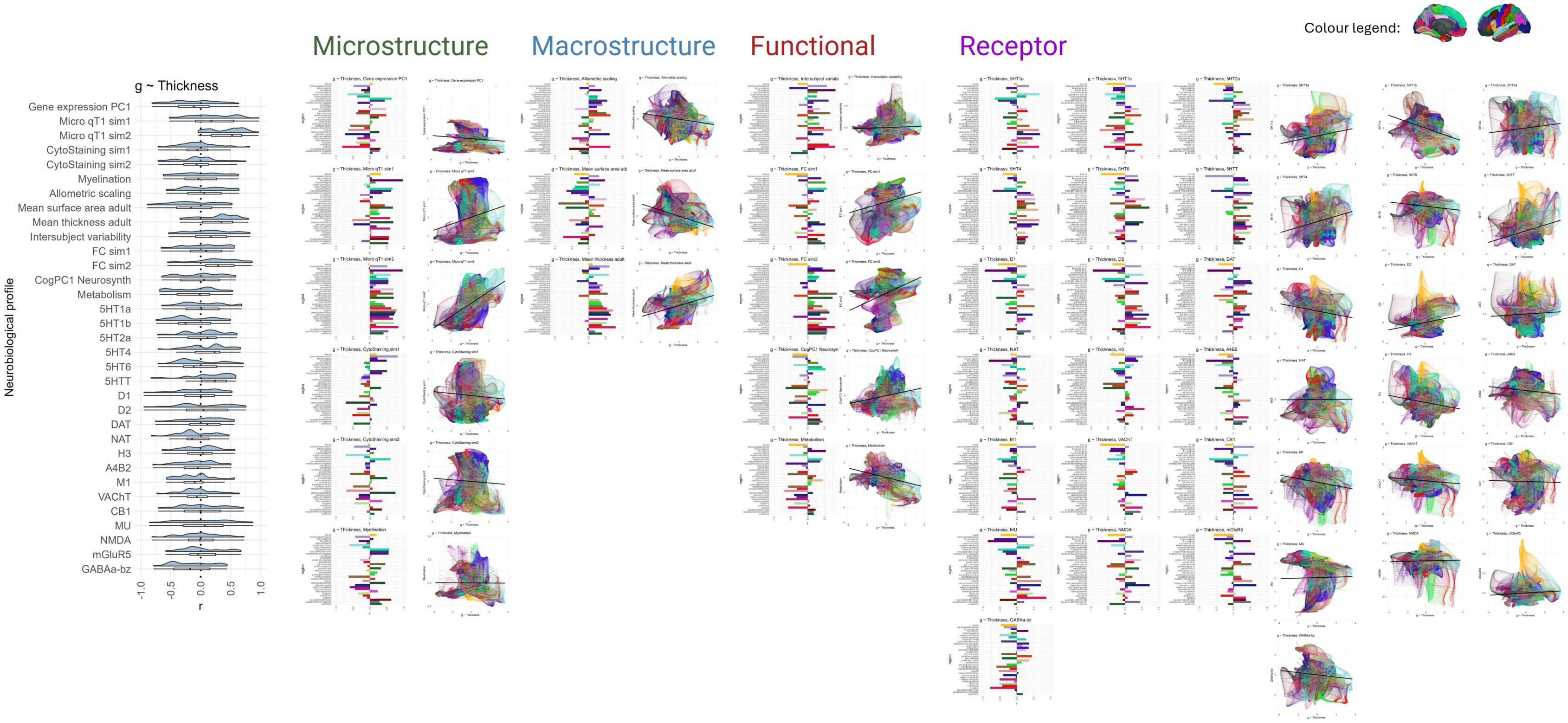

Figure S28 Within-region vertex-wise spatial correlations for ***g*-thickness and neurobiological profiles** correlations. A) summary of regional correlations, B) bar graph showing each regional correlation and scatterplots showing each correlation, coloured by Desikan-Killiany region. The data underpinning these figures are in the Supplementary Tabular Data File.

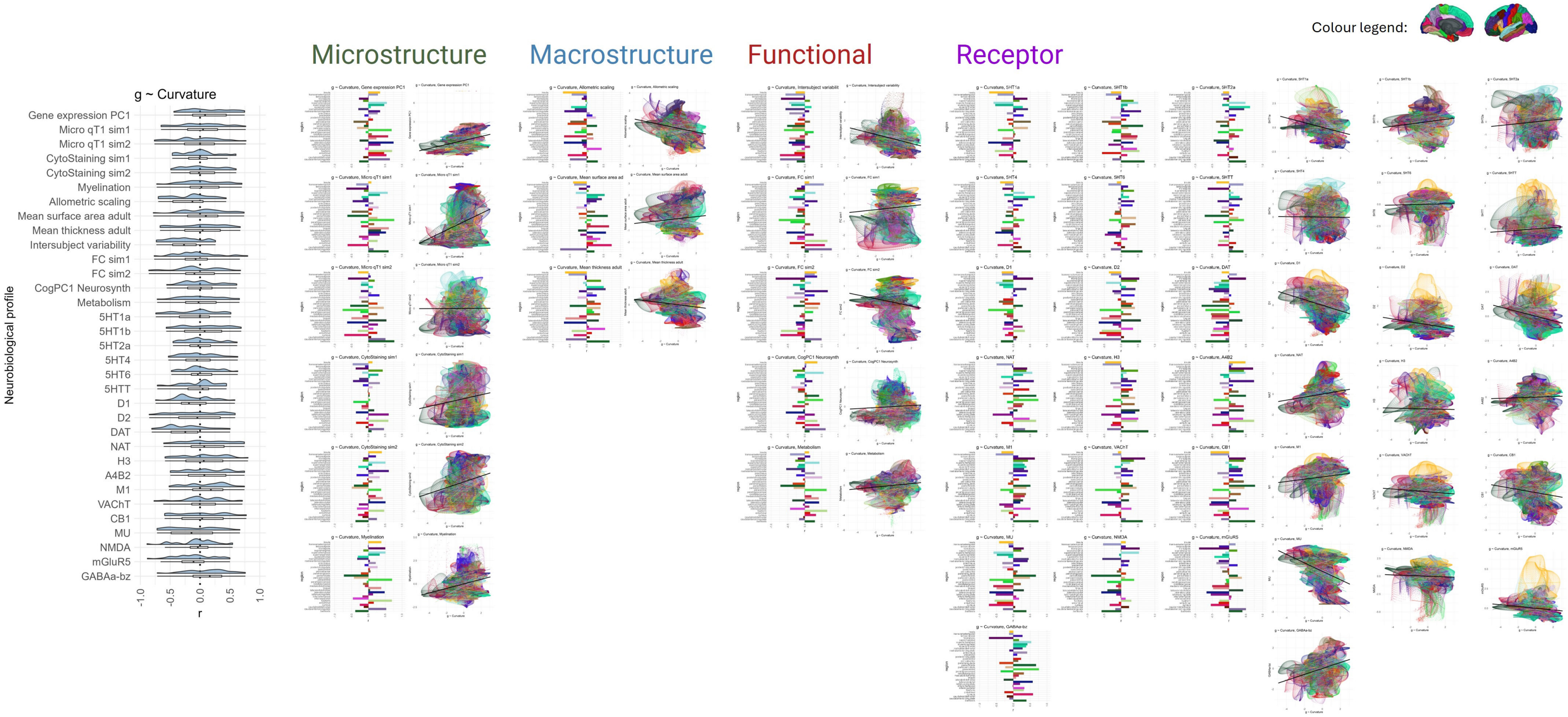

Figure S29 Within-region vertex-wise spatial correlations for ***g*-curvature and neurobiological profiles** correlations. A) summary of regional correlations, B) bar graph showing each regional correlation and scatterplots showing each correlation, coloured by Desikan-Killiany region. The data underpinning these figures are in the Supplementary Tabular Data File.

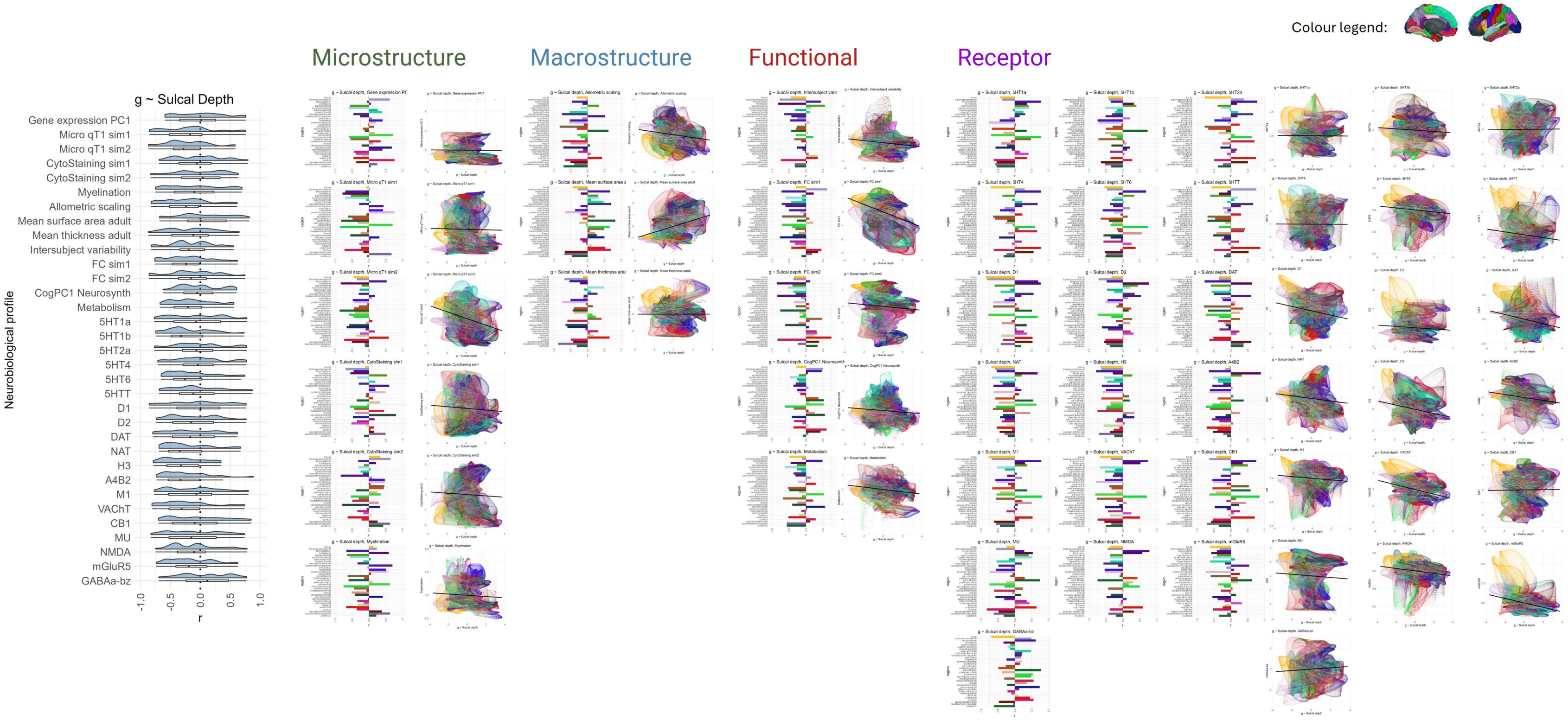

Figure S30 Within-region vertex-wise spatial correlations **for *g*-sulcal depth and neurobiological profiles** correlations. A) summary of regional correlations, B) bar graph showing each regional correlation and scatterplots showing each correlation, coloured by Desikan-Killiany region. The data underpinning these figures are in the Supplementary Tabular Data File.

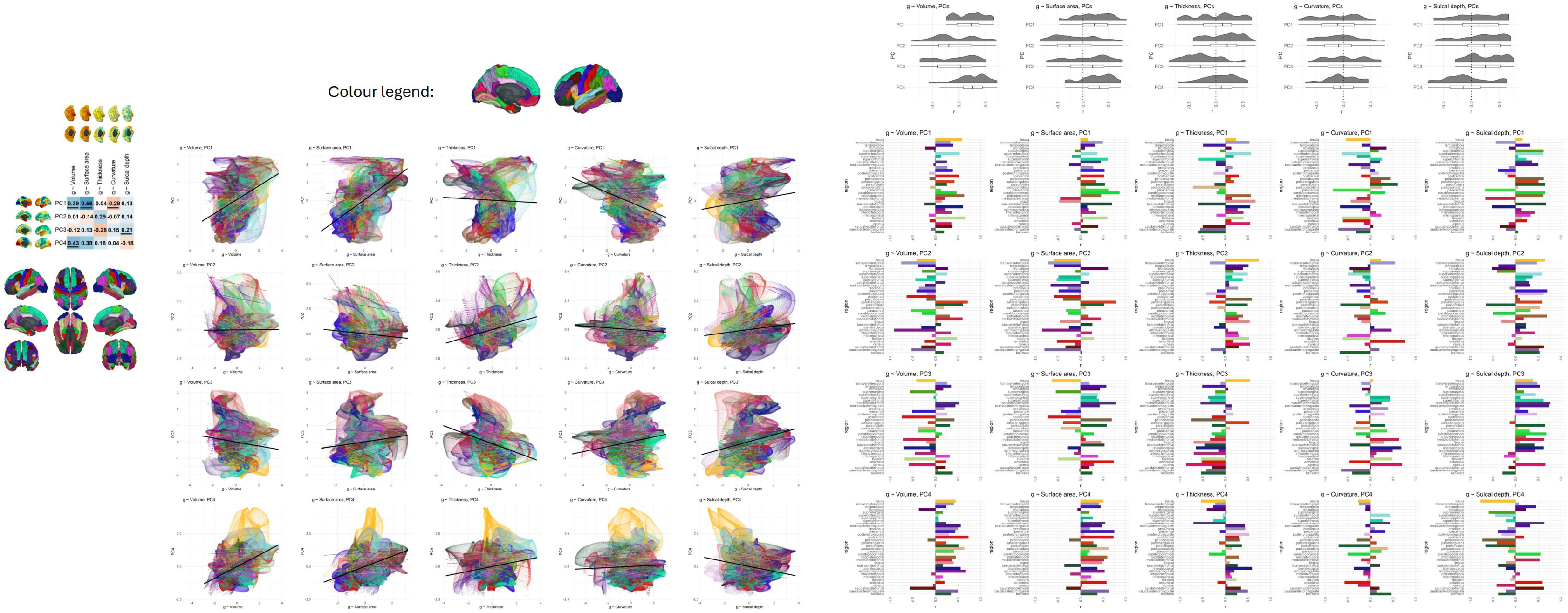

Figure S31 Regional spatial correlations for ***g* and PC** correlations. A) cortex-wide correlations, B) scatterplots showing each correlation, coloured by Desikan-Killiany region, C) summary of regional correlations, D) bar graph showing each regional correlation. The data underpinning these figures are in the Supplementary Tabular Data File.

Supplementary tables

Table S1 UK Biobank exclusions and database codes.

Exclusion criteria	Root code	Specific code
Dementia	20002	1263
Parkinson's disease	20002	1262
Stroke	20002	1081
Other chronic neurological problems	20002	1258
Other demyelinating diseases	20002	1397
Multiple sclerosis	20002	1261
Guillain–Barré syndrome	20002	1256
Brain cancer	20001	1032
Brain haemorrhage	20002	1491
Brain abscess	20002	1245
Brain aneurysm	20002	1425
Cerebral palsy	20002	1433
Encephalitis	20002	1246
Epilepsy	20002	1264
Head injury	20002	1266
Infection of nervous system	20002	1244
Ischaemic stroke	20002	1583
Meningeal cancer	20001	1031
Meningioma (benign)	20002	1659
Meningitis	20002	1247
Motor neurone disease	20002	1259
Neurological trauma	20002	1240
Spina bifida	20002	1524
Subdural haematoma	20002	1083
Subarachnoid haemorrhage	20002	1086
Transient ischaemic attack	20002	1082

Table S2 Brief descriptions of UKB cognitive tests and index codes.

Cognitive Test	Brief description	Code
Reaction time (s)	Time taken to respond in snap-type computer game	20023
Number span	Number of rounds completed (the maximum length of number string recalled)	4282
Verbal and numerical reasoning (called "fluid intelligence" in the UKB database)	Number of 13 verbal and numerical logic questions correct	20016
Trail making B (s) [1]	Time taken to complete trail B	6350
Matrix pattern (log) [2]	Number of puzzles solved	6373
Tower task	Number of puzzles solved	21003
Digit-symbol substitution [3]	Number of digit-symbol pairs matched	23324

Pairs matching [4]	Number of incorrect matches in a 6-pair classic pairs game	399
Prospective memory	Did the participant remember to ignore the instruction on their first attempt?	20018
Paired associates	Number of novel word pairs matched in recall	20197

Table S3 Brief descriptions of GenScot cognitive tests and index codes.

Cognitive Test	Brief description	Code
Matrix reasoning [2]	Number of puzzles correct	mrtotc
Verbal fluency [5]	Number of words recalled beginning with C, F and L in 3x1 minute	vftot
Mill Hill vocabulary [6]	Number of word meanings explained correctly	mhv
Digit symbol substitution [7]	Number of digit-symbol pairs matched	digsym
Logical memory [7]	Story recall score (total from immediate and delayed tests)	mema + medela

Table S4 Brief descriptions of LBC1936 cognitive tests and index codes.

Cognitive test	Brief description	Code
Matrix reasoning [8]	Number of puzzles correct	matreas_w2
Block design [8]	Number of puzzles correct	blkdes_w2
Spatial span [7]	Number of block sequences correct	spantot_w2
National adult reading test (NART) [9]	Number of words pronounced correctly	nart_w2
Weschler Test of Adult reading (WTAR) [10]	Number of words pronounced correctly	wtar_w2
Verbal fluency [11]	Number of words recalled beginning with C, F and L in 3x1 minute	vftot_w2
Verbal paired associates [7]	Number of novel word pairs matched in recall (total from immediate and delayed tests)	vpatotal_w2
Logical memory [7]	Number of story details recalled (out of a total possible of 25) Total from immediate and delayed tests	lmtotal_w2
Digit span backwards [8]	Max number of a string of numbers recalled in reverse	digback_w2
Symbol search	Number of symbols correctly detected	
Digit-symbol substitution [12]	Number of digit-symbol pairs matched	digsym_w2
Inspection time [13]	Number of correct responses – is the left or right line longer?	Ittotal_w2

Four-choice reaction time (s) [14]	Time taken to press the indicated button (out of 4 buttons)	crtmean_w2

Table S5 UKB cognitive test summary statistics, and latent cognitive ability model estimates (for all paths to the latent factor, p < .001).

Cognitive Test	*N*	*M* (*SD*)	β (*SE*)	Residual variance
Reaction time (log)	35138	6.38 (0.17)	0.22 (0.006)	0.84
Numeric memory	25720	6.76 (1.27)	-0.46 (0.006)	0.76
Fluid intelligence	34709	6.60 (2.05)	-0.70 (0.004)	0.49
Trail making B (log)	24484	6.28 (0.36)	0.59 (0.005)	0.49
Matrix pattern	25135	7.95 (2.14)	-0.58 (0.005)	0.60
Tower task	24909	9.86 (3.23)	-0.49 (0.006)	0.71
Digit-symbol substitution	25134	18.87 (5.27)	-0.44 (0.005)	0.62
Pairs matching (log)	35365	1.35 (0.63)	0.24 (0.006)	0.92
Prospective memory	35350	0.84 (0.37)	0.31 (0.006)	0.88
Paired associates	25404	7.90 (02.64)	-0.44 (0.006)	0.75

Table S6 GenScot cognitive test summary statistics and latent cognitive ability model estimates (for all paths to the latent factor, p < .001).

Cognitive Test	*N*	*M* (*SD*)	β (*SE*)	Residual variance
Matrix reasoning	1043	8.30 (2.39)	0.56 (0.029)	0.65
Verbal fluency	1043	43.10 (11.92)	0.53 (0.030)	0.71
Mill Hill vocabulary	1043	31.64 (4.07)	0.70 (0.028)	0.45
Digit symbol substitution	1043	68.77 (15.13)	0.37 (0.039)	0.64
Logical memory	1043	31.91 (7.23)	0.47 (0.030)	0.72

Table S7 LBC1936 cognitive test summary statistics, and cognitive ability model estimates (for all paths, besides the path between Verbal Memory and Cognitive ability which was fixed, all p < .001).

Cognitive test	*N*	*M (SD)*	β (*SE*)	Residual variance
Matrix reasoning	634	13.52 (4.93)	0.60 (0.03)	0.62
Block design	634	34.38 (10.01)	0.60 (0.03)	0.6
Spatial span	634	14.79 (2,72)	0.45 (0.04)	0.77
NART	634	34.66 (8.10)	0.63 (0.03)	0.57
WTAR	634	41.27 (6.94)	0.65 (0.03)	0.56
Phonemic verbal fluency	635	43.55 (12.78)	0.47 (0.04)	0.77
Verbal paired associates	623	27.57 (9.48)	0.51 (0.04)	0.7
Logical memory	635	75.03 (17.84)	0.52 (0.04)	0.71
Digit span backwards	636	7.88 (2.31)	0.55 (0.04)	0.69
Symbol search	634	24.88 (6.05)	0.58 (0.03)	0.64
Digit-symbol substitution	634	56.68 (11.79)	0.61 (0.03)	0.57
Inspection time	634	111.78 (10.95)	0.38 (0.04)	0.84
Four-choice reaction time (s)	635	0.64 (0.08)	0.39 (0.04)	0.82

Table S8 Within-domain residual variances for LBC1936 general cognitive ability model.

Cognitive test	β (*SE*)
Matrix reasoning ~~ block design	0.25 (0.05)
Matrix reasoning ~~ spatial span	0.09 (0.05)
Block design ~~ spatial span	0.20 (0.05)
NART ~~ WTAR	0.83 (0.02)
NART~~ verbal fluency	0.16 (0.05)
WTAR ~~ verbal fluency	0.16 (0.05)
Verbal paired associates ~~ logical memory	0.35 (0.04)
Verbal paired associates ~~ digit span backward	-0.04 (0.05)
Logical memory ~~ digit span backward	0.01 (0.05)
Symbol search ~~ digit symbol	0.40 (0.04)
Symbol search ~~ inspection time	0.17 (0.04)
Symbol search ~~ choice reaction time	0.31 (0.04)
Digit symbol ~~ inspection time	0.22 (0.04)
Digit symbol ~~ choice reaction time	0.36 (0.04)
Inspection time ~~ choice reaction time	0.25 (0.04)

Table S9 Model fits for the latent cognitive ability models.

Cohort	X^2	*df*	CFI	TLI	RMSEA	SRMR
UKB	2417	34	0.953	0.913	0.042	0.026
GenScot	1170	20	0.972	0.887	0.079	0.019
LBC1936	168	35	0.966	0.930	0.061	0.037

Table S10 Between-cohort spatial correlations r for vertex-wise mean profiles for each measure. All $p < 2.2x10^{-16}$.

Measure	*r* LBC-GenScot	*r* GenScot-UKB	*r* UKB-LBC
Volume	0.986	0.966	0.961
Surface area	0.990	0.977	0.969
Thickness	0.934	0.907	0.843
Curvature	0.997	0.968	0.972
Sulcal depth	0.998	0.993	0.990

Table S11 Spatial correlations (Pearson's r) between absolute value g-associations (β) for the 5 vertex-wise measures (all $p < 2.2x10^{-16}$).

		Volume	Surface area	Thickness	Curvature
g	Volume	1			
	Surface area	0.653	1		
	Thickness	0.413	-0.077	1	
	Curvature	-0.093	0.113	-0.236	1
	Sulcal depth	0.032	0.241	-0.151	0.182
Age	Volume	1			
	Surface area	0.550	1		
	Thickness	0.380	-0.350	1	
	Curvature	-0.248	0.006	-0.127	1
	Sulcal depth	-0.064	0.019	-0.182	0.308
Sex	Volume	1			
	Surface area	0.769	1		
	Thickness	-0.426	-0.166	1	
	Curvature	-0.097	0.163	0.203	1
	Sulcal depth	0.126	0.207	0.024	0.237

Table S12 Brief descriptions of the strongest meta-analysed vertex-wise mappings

	g		Age		Sex	
Measure	+ve	-ve	+ve	-ve	+ve (male > female)	-ve (female > male)
Volume	Superior frontal, medial frontal, lateral temporal, parietal	-		Lateral temporal, medial frontal, ventrolateral prefrontal	Insula, fusiform gyrus, superior frontal	

Surface area	Superior frontal, medial frontal, medial orbitofrontal, anterior cingulate, lateral temporal, parietal regions	-		Lateral temporal	Insula, fusiform, frontal	
Thickness	Temporal pole, entorhinal cortex, precentral	Anterior cingulate, medial orbitofrontal, medial occipital		Dorsolateral prefrontal, superior temporal, fusiform gyrus	Lateral temporal, medial orbitofrontal	Superior frontal and parietal regions
Curvature	Medial frontal, medial occipital	Anterior cingulate	Insula	Medial occipital, temporal, superior frontal gyrus	Precentral, medial frontal, medial occipital	Anterior cingulate
Sulcal depth	Medial frontal, temporal pole	Cingulate, hippocampal gyrus, parieto-frontal regions	Anterior cingulate, medial frontal, insula	Medial orbitofrontal, posterior cingulate, lateral orbitofrontal	Medial frontal, medial occipital	Insula, caudate anterior, posterior and isthmus cingulate, lateral temporal

Table S13 Descriptive statistics for the multi-scale cortical profiles. The fsaverage surface is not symmetrical, so there is no direct correspondence of the position of vertices on left and right hemispheres. Therefore, to calculate the left and right spatial correlations, we took the mean of the values (for each measure) at each of the 68 Desikan-Killiany regions (34 per region) and correlated the left and right values). For subcortical measures, out of the 42 structures, 26 were part of left/right pairings (13 left and 13 right) – it is these that are correlated in the L vs R column here. The cytoarchitectural, functional and microstructural eigenvectors are in the units they come in through BigBrainWarp. The neurotransmitter receptor density profiles are scaled here.

Measure	*N* regions/vertices	Measure	M	SD	Min	Max	Range	Skew	Kurtosis	L vs R (*r*)	% +ve	% FDR Q < .05
g_ global_subcortical	42	β	0.09	0.07	-0.06	0.19	0.25	-0.67	-0.57	0.970	-	-
Age_global_subcortical	42	β	-0.11	0.28	-0.43	0.49	0.92	0.90	-0.79	0.993	-	-
Sex_global_subcortical	42	β	0.30	0.12	0.03	0.49	0.46	-0.42	-0.69	0.985	-	-
g_Volume	298790	β	0.09	0.02	0.00	0.17	0.17	0.50	0.32	0.870	100	99
g_Surface area	298790	β	0.09	0.02	0.01	0.15	0.14	0.37	-0.15	0.855	100	100
g_Thickness	298790	β	0.03	0.03	-0.08	0.13	0.21	0.21	1.18	0.940	89	27
g_Curvature	298790	β	0.02	0.02	-0.10	0.09	0.19	-0.43	1.44	0.948	78	40
g_Sulcal depth	298790	β	0.00	0.03	-0.12	0.13	0.24	0.14	1.00	0.950	54	26
Age_Volume	298790	β	-0.16	0.05	-0.32	0.03	0.36	0.28	0.37	0.918	0	92
Age_Surface area	298790	β	-0.07	0.05	-0.21	0.11	0.32	0.24	0.34	0.855	8	59
Age_Thickness	298790	β	-0.20	0.07	-0.36	0.06	0.42	0.73	0.32	0.949	1	91
Age_Curvature	298790	β	-0.03	0.05	-0.29	0.17	0.46	-0.65	2.57	0.948	20	55
Age_Sulcal depth	298790	β	0.01	0.06	-0.21	0.23	0.44	-0.08	0.82	0.881	57	45
Sex_Volume	298790	β	0.26	0.07	0.01	0.45	0.44	-0.15	-0.29	0.948	100	99
Sex_Surface area	298790	β	0.31	0.05	0.09	0.46	0.38	0.11	-0.18	0.927	100	100
Sex_Thickness	298790	β	-0.03	0.06	-0.23	0.15	0.39	0.19	-0.02	0.938	28	61
Sex_Curvature	298790	β	0.06	0.08	-0.21	0.30	0.52	-0.20	-0.19	0.867	78	62
Sex_Sulcal depth	298790	B	0.02	0.08	-0.24	0.25	0.49	-0.08	-0.20	0.936	62	69
Allometric scaling	296637	β	0.60	0.08	0.30	0.82	0.52	0.04	-0.49	0.983	100	-
Metabolism	296637	Principal component	0.00	1.63	-9.13	5.60	14.74	-0.52	1.51	0.986	52	-
Cytoarchitecture 1	298790	Eigenvector	0.00	0.31	-0.52	0.69	1.21	0.26	-1.01	0.954	47	-
Cytoarchitecture 2	298790	Eigenvector	0.04	0.21	-0.44	0.50	0.94	-0.12	-0.77	0.963	58	-

Functional 1	298790	Eigenvector	0.02	0.28	-0.88	0.52	1.40	-0.95	0.11	0.982	63	-
Functional 2	298790	Eigenvector	0.01	0.15	-0.37	0.26	0.62	-0.62	-0.61	0.994	62	-
Microstructure 1	298790	Eigenvector	0.00	0.07	-0.10	0.16	0.26	0.54	-0.90	0.982	43	-
Microstructure 2	298790	Eigenvector	0.00	0.01	-0.02	0.03	0.05	-0.04	-0.77	0.843	49	-
5HT1a	298790	Receptor density (z)	0.00	1.00	-2.27	4.37	6.64	0.72	1.05	0.991	46	-
5HT1b	298790	Receptor density (z)	0.00	1.00	-3.22	3.79	7.01	-0.11	0.52	0.991	51	-
5HT2a	298790	Receptor density (z)	0.00	1.00	-2.42	2.87	5.29	0.31	-0.66	0.956	47	-
5HT4	298790	Receptor density (z)	0.00	1.00	-2.14	3.08	5.22	0.46	-0.46	0.969	45	-
5HT6	298790	Receptor density (z)	0.00	1.00	-4.83	2.88	7.71	-1.06	2.02	0.943	58	-
5HTT	298790	Receptor density (z)	0.00	1.00	-1.52	4.32	5.84	1.33	1.83	0.952	39	-
D1	298790	Receptor density (z)	0.00	1.00	-2.78	3.18	5.96	-0.09	-0.38	0.962	54	-
D2	298790	Receptor density (z)	0.00	1.00	-1.66	5.47	7.13	1.26	2.20	0.962	39	-
DAT	298790	Receptor density (z)	0.00	1.00	-2.46	4.40	6.85	0.63	1.22	0.993	49	-
NAT	298790	Receptor density (z)	0.00	1.00	-3.34	3.18	6.52	-0.02	0.32	0.982	52	-
H3	298790	Receptor density (z)	0.00	1.00	-2.35	3.86	6.21	0.78	0.50	0.818	44	-
A4B2	298790	Receptor density (z)	0.00	1.00	-4.27	3.45	7.72	-0.47	0.70	0.980	52	-
M1	298790	Receptor density (z)	0.00	1.00	-4.33	2.36	6.69	-0.85	0.94	0.993	56	-
VAChT	298790	Receptor density (z)	0.00	1.00	-4.42	3.71	8.13	0.01	0.61	0.972	50	-
CB1	298790	Receptor density (z)	0.00	1.00	-2.86	2.51	5.37	-0.17	-0.60	0.991	51	-
MU	298790	Receptor density (z)	0.00	1.00	-2.44	1.93	4.37	-0.67	-0.22	0.972	58	-
NMDA	298790	Receptor density (z)	0.00	1.00	-6.47	2.98	9.45	-1.20	4.93	0.872	56	-
mGluR5	298790	Receptor density (z)	0.00	1.00	-1.80	6.80	8.60	2.41	9.59	0.996	42	-
GABAa-bz	298790	Receptor density (z)	0.00	1.00	-3.41	3.10	6.50	-0.19	0.34	0.997	54	-

Table S14 Descriptive statistics of estimates within each cohort, and the between-cohort relative correlations (Pearson's r) of the 46 global and subcortical estimates (all $p < .05$). Note, the summary estimates are for the standardised betas.

	LBC1936			GenScot			UKB			Between-cohort relative consistency (*r*)		
	Mean (SD)	Min	Max	Mean (SD)	Min	Max	Mean (SD)	Min	Max	LBC1936-GenScot	GenScot-UKB	UKB-LBC1936
g	0.127 (0.116)	-0.139	0.283	0.091 (0.057)	-0.051	0.174	0.072 (0.045)	-0.025	0.133	0.724	0.858	0.820
Age	-	-	-	-0.134 (0.270)	-0.401	0.460	-0.094 (0.283)	-0.452	0.502	-	0.967	-
Sex	0.260 (0.121)	-0.016	0.468	0.275 (0.122)	0.074	0.469	0.345 (0.142)	0.016	0.568	0.795	0.914	0.825
Allometry	0.581 (0.094)	0.232	0.843	0.593 (0.096)	0.299	0.853	0.599 (0.088)	0.012	0.822	0.754	0.726	0.779

Table S15 Meta-analysis outcomes for ***g*** ~ global and subcortical brain structure volume associations (ordered by decreasing beta values).

Region	β	*SE*	*z*	*p*	*FDR Q*	Cochrane's *Q*	*p* (*Q*)	I^2
Total grey matter	0.191	0.044	4.319	0.000	0.000	26.144	0.000	0.005
Cerebral GM	0.183	0.042	4.372	0.000	0.000	24.207	0.000	0.005
Cerebral WM	0.180	0.043	4.174	0.000	0.000	22.571	0.000	0.005
TBV	0.174	0.037	4.729	0.000	0.000	16.617	0.000	0.004
Subcortical GM	0.156	0.024	6.420	0.000	0.000	8.029	0.018	0.001
Brain stem	0.148	0.028	5.243	0.000	0.000	12.785	0.002	0.002
Left ventral DC	0.148	0.035	4.231	0.000	0.000	12.320	0.002	0.003
Right Cerebellum GM	0.146	0.032	4.592	0.000	0.000	10.217	0.006	0.003
Right hippocampus	0.141	0.028	5.016	0.000	0.000	9.691	0.008	0.002
Right ventral DC	0.139	0.027	5.245	0.000	0.000	8.395	0.015	0.002
Left Cerebellum GM	0.133	0.021	6.312	0.000	0.000	5.356	0.069	0.001
CC posterior	0.131	0.039	3.332	0.001	0.001	16.077	0.000	0.004
CC anterior	0.127	0.051	2.506	0.012	0.017	19.777	0.000	0.007
Left Cerebellum WM	0.123	0.022	5.678	0.000	0.000	5.942	0.051	0.001
CC mid posterior	0.122	0.053	2.284	0.022	0.029	24.455	0.000	0.008
Left hippocampus	0.117	0.011	10.370	0.000	0.000	2.020	0.364	0.000
Right pallidum	0.115	0.031	3.680	0.000	0.000	7.803	0.020	0.002
Left pallidum	0.113	0.040	2.798	0.005	0.007	10.759	0.005	0.004
Left thalamus	0.111	0.004	24.822	0.000	0.000	1.950	0.377	0.000
Right putamen	0.111	0.029	3.786	0.000	0.000	11.547	0.003	0.002
Left putamen	0.111	0.028	4.019	0.000	0.000	10.393	0.006	0.002
Right thalamus	0.110	0.004	26.765	0.000	0.000	1.336	0.513	0.000
CC mid anterior	0.107	0.054	1.985	0.047	0.056	22.577	0.000	0.008
CC central	0.106	0.063	1.689	0.091	0.101	30.329	0.000	0.011
Right Cerebellum WM	0.102	0.020	5.100	0.000	0.000	4.562	0.102	0.001
Right amygdala	0.095	0.004	21.707	0.000	0.000	1.862	0.394	0.000
Right accumbens	0.093	0.029	3.243	0.001	0.002	6.352	0.042	0.002
Left amygdala	0.090	0.011	8.533	0.000	0.000	2.308	0.315	0.000
Left caudate	0.075	0.019	3.945	0.000	0.000	4.180	0.124	0.001
Left accumbens	0.067	0.005	14.578	0.000	0.000	3.582	0.167	0.000
Right caudate	0.063	0.005	13.529	0.000	0.000	1.585	0.453	0.000
4th ventricle	0.035	0.005	7.426	0.000	0.000	3.583	0.167	0.000
Optic chiasm	0.014	0.004	3.123	0.002	0.003	0.159	0.923	0.000
CSF	0.012	0.041	0.305	0.760	0.779	10.487	0.005	0.004
Left choroid plexus	0.011	0.007	1.725	0.085	0.096	2.176	0.337	0.000
Right choroid plexus	0.009	0.004	2.171	0.030	0.038	0.069	0.966	0.000
Right lateral ventricle	0.001	0.005	0.182	0.856	0.856	1.595	0.451	0.000
Left lateral ventricle	-0.002	0.005	-0.551	0.581	0.611	2.527	0.283	0.000
3rd ventricle	-0.026	0.016	-1.645	0.100	0.108	4.074	0.130	0.000
Left inferior lateral ventricle	-0.028	0.004	-6.383	0.000	0.000	1.056	0.590	0.000
Right inferior lateral ventricle	-0.057	0.027	-2.130	0.033	0.041	9.015	0.011	0.002
WM hypointensities	-0.063	0.032	-1.981	0.048	0.056	9.619	0.008	0.002

Table S16 Meta-analysis outcomes for global and subcortical brain structure volume ~ **age** associations (ordered by decreasing beta values).

Region	β	*SE*	*z*	*p*	*FDR Q*	Cochrane's *Q*	*p* (*Q*)	I^2
Left accumbens	-0.431	0.024	-17.823	0.000	0.000	4.781	0.029	0.001
Right accumbens	-0.375	0.004	-92.348	0.000	0.000	0.284	0.594	0.000
Left hippocampus	-0.364	0.004	-90.425	0.000	0.000	0.022	0.881	0.000
Right hippocampus	-0.363	0.004	-89.332	0.000	0.000	0.714	0.398	0.000
Left amygdala	-0.342	0.036	-9.608	0.000	0.000	7.419	0.006	0.002
Left thalamus	-0.336	0.024	-13.798	0.000	0.000	4.508	0.034	0.001
CC mid anterior	-0.333	0.004	-75.824	0.000	0.000	0.102	0.750	0.000
Right thalamus	-0.328	0.014	-24.056	0.000	0.000	1.981	0.159	0.000
Right ventral DC	-0.327	0.039	-8.372	0.000	0.000	11.414	0.001	0.003
Total grey matter	-0.319	0.036	-8.883	0.000	0.000	12.571	0.000	0.002
CC central	-0.315	0.018	-17.378	0.000	0.000	2.258	0.133	0.000
Cerebral WM	-0.307	0.030	-10.158	0.000	0.000	8.086	0.004	0.002
Left putamen	-0.306	0.060	-5.070	0.000	0.000	24.243	8.49E-07	0.007
Right putamen	-0.306	0.061	-4.978	0.000	0.000	26.496	2.64E-07	0.007
CC mid posterior	-0.301	0.061	-4.962	0.000	0.000	21.761	3.09E-06	0.007
Right amygdala	-0.287	0.004	-69.675	0.000	0.000	0.447	0.504	0.000
Left ventral DC	-0.285	0.015	-19.026	0.000	0.000	1.989	0.158	0.000
Subcortical GM	-0.281	0.062	-4.545	0.000	0.000	31.543	1.95E-08	0.007
TBV	-0.250	0.051	-4.861	0.000	0.000	14.376	0.000	0.005
Left Cerebellum GM	-0.231	0.065	-3.530	0.000	0.000	32.031	1.52E-08	0.008
Left Cerebellum WM	-0.228	0.072	-3.181	0.001	0.002	26.952	2.09E-07	0.010
Right Cerebellum WM	-0.224	0.052	-4.313	0.000	0.000	13.029	0.000	0.005
Cerebral GM	-0.220	0.004	-54.317	0.000	0.000	0.503	0.478	0.000
Right Cerebellum GM	-0.219	0.047	-4.626	0.000	0.000	18.329	1.86E-05	0.004
CC anterior	-0.211	0.054	-3.907	0.000	0.000	14.619	0.000	0.005
Left pallidum	-0.152	0.005	-33.826	0.000	0.000	0.003	0.960	0.000
Brain stem	-0.142	0.035	-4.087	0.000	0.000	7.097	0.008	0.002
Right pallidum	-0.137	0.013	-10.335	0.000	0.000	1.491	0.222	0.000
CC posterior	-0.090	0.066	-1.366	0.172	0.176	19.031	1.29E-05	0.008
Left caudate	-0.082	0.057	-1.445	0.148	0.156	15.094	0.000	0.006
Right caudate	-0.071	0.075	-0.950	0.342	0.342	26.200	3.08E-07	0.011
4th ventricle	0.115	0.013	8.551	0.000	0.000	1.460	0.227	0.000
CSF	0.219	0.004	49.383	0.000	0.000	0.063	0.802	0.000
Optic chiasm	0.227	0.004	51.311	0.000	0.000	0.321	0.571	0.000
Right inferior lateral ventricle	0.283	0.064	4.404	0.000	0.000	20.098	7.36E-06	0.008
Left choroid plexus	0.327	0.067	4.902	0.000	0.000	26.307	2.91E-07	0.009
Left inferior lateral ventricle	0.354	0.035	10.135	0.000	0.000	7.104	0.008	0.002
Right choroid plexus	0.358	0.043	8.394	0.000	0.000	11.995	0.001	0.003
Left lateral ventricle	0.364	0.015	23.712	0.000	0.000	1.890	0.169	0.000
Right lateral ventricle	0.378	0.017	22.423	0.000	0.000	2.150	0.143	0.000
3rd ventricle	0.397	0.004	106.683	0.000	0.000	0.166	0.684	0.000
WM hypointensities	0.487	0.020	23.985	0.000	0.000	3.368	0.066	0.001

Table S17 Meta-analysis outcomes global and subcortical brain structure volume ~ **sex** associations (ordered by decreasing beta values).

Region	β	*SE*	*z*	*p*	*FDR Q*	Cochrane's *Q*	*p* (*Q*)	I^2
Total grey matter	0.490	0.043	11.475	0.000	0.000	46.446	0.000	0.005
Subcortical GM	0.466	0.054	8.569	0.000	0.000	48.029	0.000	0.008
TBV	0.466	0.053	8.743	0.000	0.000	51.550	0.000	0.008
Cerebral WM	0.462	0.045	10.214	0.000	0.000	27.210	0.000	0.006
Cerebral GM	0.455	0.042	10.918	0.000	0.000	36.697	0.000	0.005
Brain stem	0.447	0.034	12.968	0.000	0.000	15.692	0.000	0.003
Right Cerebellum GM	0.439	0.038	11.505	0.000	0.000	29.706	0.000	0.004
Left ventral DC	0.432	0.040	10.743	0.000	0.000	18.679	0.000	0.004
Right ventral DC	0.423	0.041	10.213	0.000	0.000	22.223	0.000	0.005
Left Cerebellum GM	0.420	0.039	10.793	0.000	0.000	21.197	0.000	0.004
3rd ventricle	0.392	0.033	11.868	0.000	0.000	9.580	0.008	0.003
Left amygdala	0.366	0.018	19.988	0.000	0.000	4.112	0.128	0.001
Right amygdala	0.350	0.050	7.002	0.000	0.000	41.785	0.000	0.007
Left choroid plexus	0.347	0.022	15.447	0.000	0.000	5.002	0.082	0.001
Right choroid plexus	0.345	0.029	11.914	0.000	0.000	10.724	0.005	0.002
Right thalamus	0.344	0.056	6.116	0.000	0.000	59.414	0.000	0.009
Right putamen	0.339	0.043	7.859	0.000	0.000	26.758	0.000	0.005
Left putamen	0.338	0.048	7.088	0.000	0.000	30.591	0.000	0.006
CSF	0.336	0.017	20.136	0.000	0.000	3.324	0.190	0.000
Left pallidum	0.328	0.051	6.462	0.000	0.000	31.327	0.000	0.007
Left thalamus	0.323	0.043	7.581	0.000	0.000	27.543	0.000	0.005
Right pallidum	0.311	0.069	4.497	0.000	0.000	40.437	0.000	0.014
Left hippocampus	0.297	0.031	9.444	0.000	0.000	11.803	0.003	0.002
Right hippocampus	0.297	0.023	12.995	0.000	0.000	6.072	0.048	0.001
Left inferior lateral ventricle	0.285	0.020	14.590	0.000	0.000	4.337	0.114	0.001
Right lateral ventricle	0.274	0.005	59.872	0.000	0.000	2.519	0.284	0.000
Right inferior lateral ventricle	0.268	0.041	6.600	0.000	0.000	9.980	0.007	0.004
Left lateral ventricle	0.267	0.004	62.887	0.000	0.000	2.459	0.292	0.000
4th ventricle	0.260	0.026	9.976	0.000	0.000	5.696	0.058	0.001
Right caudate	0.248	0.040	6.280	0.000	0.000	17.563	0.000	0.004
Left caudate	0.247	0.031	7.996	0.000	0.000	9.699	0.008	0.002
Optic chiasm	0.229	0.064	3.566	0.000	0.000	45.542	0.000	0.012
Right accumbens	0.192	0.051	3.794	0.000	0.000	37.051	0.000	0.007
Right Cerebellum WM	0.189	0.029	6.562	0.000	0.000	8.804	0.012	0.002
Left Cerebellum WM	0.178	0.044	4.031	0.000	0.000	23.445	0.000	0.005
Left accumbens	0.169	0.047	3.564	0.000	0.000	30.080	0.000	0.006
WM hypointensities	0.136	0.023	6.026	0.000	0.000	5.299	0.071	0.001
CC posterior	0.120	0.005	24.525	0.000	0.000	1.245	0.537	0.000
CC anterior	0.115	0.042	2.741	0.006	0.006	11.039	0.004	0.004
CC mid anterior	0.059	0.013	4.424	0.000	0.000	3.780	0.151	0.000
CC mid posterior	0.058	0.007	8.899	0.000	0.000	1.591	0.451	0.000
CC central	0.028	0.018	1.556	0.120	0.120	3.409	0.182	0.000

Table S18 **Between-cohort age moderation outcomes** for meta-analysis of ***g*** ~ global and subcortical brain structure volume associations (ordered by decreasing beta values, structures for which *FDR Q* < .05 are in bold font).

Region	β	*SE*	*z*	*p*	*FDR Q*	Cochrane's *Q*	*p* (*Q*)	I^2
CC central	0.015	0.008	1.894	0.058	0.321	13.890	0.000	0.004
CC mid anterior	0.012	0.007	1.764	0.078	0.327	10.980	0.001	0.003
CC anterior	0.012	0.006	1.964	0.050	0.321	7.935	0.005	0.002
CC mid posterior	0.012	0.008	1.541	0.123	0.351	14.066	0.000	0.004
**Left pallidum**	**0.011**	**0.003**	**3.205**	**0.001**	**0.028**	**0.488**	**0.485**	**0.000**
Total GM	0.010	0.007	1.450	0.147	0.363	17.900	0.000	0.003
Cerebral WM	0.009	0.007	1.330	0.184	0.386	14.642	0.000	0.004
Cerebral GM	0.009	0.007	1.235	0.217	0.396	18.346	0.000	0.004
Right pallidum	0.009	0.004	2.207	0.027	0.287	1.850	0.174	0.000
Right accumbens	0.009	0.003	2.468	0.014	0.190	0.262	0.609	0.000
Left ventral DC	0.008	0.005	1.669	0.095	0.333	6.221	0.013	0.001
TBV	0.008	0.005	1.527	0.127	0.351	7.914	0.005	0.002
Right cerebellum GM	0.008	0.004	1.781	0.075	0.327	5.051	0.025	0.001
CC posterior	0.007	0.008	0.952	0.341	0.530	12.227	0.000	0.004
Left accumbens	0.006	0.003	1.880	0.060	0.321	0.047	0.828	0.000
Right ventral DC	0.006	0.005	1.338	0.181	0.386	5.102	0.024	0.001
Left cerebellum GM	0.006	0.004	1.684	0.092	0.333	2.084	0.149	0.000
Subcortical GM	0.005	0.005	1.172	0.241	0.405	5.852	0.016	0.001
Right Cerebellum WM	0.005	0.004	1.260	0.208	0.396	2.436	0.119	0.001
Right hippocampus	0.005	0.006	0.828	0.408	0.591	8.143	0.004	0.002
Right putamen	0.005	0.006	0.703	0.482	0.614	10.637	0.001	0.003
Right amygdala	0.004	0.003	1.355	0.176	0.386	0.027	0.870	0.000
Left pallidum	0.004	0.006	0.551	0.582	0.679	9.879	0.002	0.003
Left amygdala	0.003	0.004	0.729	0.466	0.612	1.707	0.191	0.000
Left thalamus	0.003	0.003	0.780	0.435	0.609	1.363	0.243	0.000
Left cerebellum WM	0.002	0.005	0.431	0.667	0.737	5.751	0.016	0.002
Brain stem	0.002	0.007	0.319	0.750	0.807	12.652	0.000	0.003
Right thalamus	0.002	0.003	0.563	0.573	0.679	1.021	0.312	0.000
Left hippocampus	0.000	0.004	0.070	0.944	0.944	2.020	0.155	0.000
Optic chiasm	0.000	0.004	-0.092	0.926	0.944	0.151	0.698	0.000
Right choroid plexus	-0.001	0.003	-0.167	0.867	0.911	0.041	0.840	0.000
Left inferior lateral ventricle	-0.002	0.003	-0.660	0.509	0.629	0.620	0.431	0.000
Right caudate	-0.003	0.004	-0.732	0.464	0.612	1.039	0.308	0.000
Right inferior lateral ventricle	-0.003	0.006	-0.465	0.642	0.728	8.330	0.004	0.003
Left caudate	-0.004	0.004	-0.850	0.395	0.591	2.860	0.091	0.001
Right lateral ventricle	-0.004	0.003	-1.144	0.253	0.408	0.286	0.593	0.000
Left choroid plexus	-0.004	0.003	-1.206	0.228	0.398	0.721	0.396	0.000
Left lateral ventricle	-0.005	0.003	-1.499	0.134	0.351	0.280	0.597	0.000
3rd ventricle	-0.005	0.003	-1.604	0.109	0.351	1.330	0.249	0.000
4th ventricle	-0.007	0.004	-1.872	0.061	0.321	0.079	0.779	0.000
WM hypointensities	-0.007	0.006	-1.302	0.193	0.386	6.308	0.012	0.002
**CSF**	**-0.011**	**0.003**	**-3.213**	**0.001**	**0.028**	**0.167**	**0.683**	**0.000**

Table S19 **Between-cohort age moderation outcomes** for meta-analysis of global and subcortical brain structure volume ~ **sex** associations (ordered by decreasing beta values, FDR Q < .05 associations are in bold font).

Region	B	*SE*	*z*	*p*	*FDR Q*	Cochrane's *Q*	*p* (*Q*)	I^2
Right inferior lateral ventricle	0.011	0.004	3.144	0.002	0.070	0.293	0.589	0.000
Optic chiasm	0.010	0.013	0.753	0.451	0.885	37.910	0.000	0.015
3rd ventricle	0.009	0.003	2.894	0.004	0.080	1.177	0.278	0.000
Left inferior lateral ventricle	0.007	0.004	2.043	0.041	0.515	0.322	0.570	0.000
Left accumbens	0.007	0.010	0.700	0.484	0.885	23.248	0.000	0.008
Right lateral ventricle	0.006	0.004	1.607	0.108	0.649	0.030	0.863	0.000
Left lateral ventricle	0.005	0.004	1.472	0.141	0.705	0.437	0.509	0.000
WM hypointensities	0.005	0.005	1.025	0.305	0.885	3.118	0.077	0.001
TBV	0.004	0.013	0.266	0.790	0.889	52.072	0.000	0.015
Right amygdala	0.002	0.013	0.152	0.879	0.928	41.708	0.000	0.013
Left amygdala	0.000	0.005	0.036	0.972	0.992	4.541	0.033	0.001
Right accumbens	0.000	0.013	0.010	0.992	0.992	37.107	0.000	0.014
CSF	-0.001	0.005	-0.147	0.883	0.928	3.299	0.069	0.001
CC posterior	-0.002	0.004	-0.432	0.666	0.885	1.394	0.238	0.000
Right Cerebellum WM	-0.002	0.007	-0.306	0.759	0.886	9.262	0.002	0.003
Right hippocampus	-0.002	0.006	-0.392	0.695	0.885	6.589	0.010	0.002
Right Cerebellum GM	-0.003	0.010	-0.326	0.745	0.886	30.782	0.000	0.008
CC mid posterior	-0.003	0.004	-0.794	0.427	0.885	0.823	0.364	0.000
Right choroid plexus	-0.003	0.007	-0.494	0.622	0.885	10.429	0.001	0.003
Right thalamus	-0.003	0.014	-0.247	0.805	0.889	60.073	0.000	0.017
Total grey matter	-0.004	0.011	-0.344	0.731	0.886	46.853	0.000	0.010
Left hippocampus	-0.004	0.008	-0.495	0.621	0.885	12.092	0.001	0.004
Right caudate	-0.004	0.009	-0.430	0.667	0.885	17.350	0.000	0.007
Left caudate	-0.004	0.007	-0.591	0.555	0.885	9.246	0.002	0.003
Left Cerebellum WM	-0.004	0.011	-0.410	0.682	0.885	23.913	0.000	0.009
Right putamen	-0.005	0.010	-0.443	0.658	0.885	27.327	0.000	0.009
Left thalamus	-0.005	0.010	-0.451	0.652	0.885	28.376	0.000	0.009
Cerebral WM	-0.005	0.010	-0.461	0.645	0.885	37.239	0.000	0.009
CC central	-0.005	0.004	-1.175	0.240	0.875	1.609	0.205	0.000
Left putamen	-0.006	0.011	-0.527	0.598	0.885	30.545	0.000	0.010
Brain stem	-0.006	0.007	-0.860	0.390	0.885	14.411	0.000	0.004
Left Cerebellum GM	-0.007	0.008	-0.804	0.421	0.885	20.881	0.000	0.005
Left choroid plexus	-0.007	0.004	-1.779	0.075	0.527	1.530	0.216	0.000
4th ventricle	-0.007	0.005	-1.436	0.151	0.705	2.834	0.092	0.001
Left pallidum	-0.007	0.012	-0.606	0.544	0.885	29.964	0.000	0.011
CC mid anterior	-0.007	0.004	-1.871	0.061	0.515	0.674	0.412	0.000
Right ventral DC	-0.008	0.008	-0.963	0.336	0.885	19.884	0.000	0.005
Left ventral DC	-0.009	0.007	-1.150	0.250	0.875	15.513	0.000	0.004
Subcortical GM	-0.009	0.012	-0.777	0.437	0.885	46.622	0.000	0.011
Cerebral GM	-0.009	0.009	-1.032	0.302	0.885	23.283	0.000	0.006
CC anterior	-0.011	0.006	-1.905	0.057	0.515	4.645	0.031	0.002
Right pallidum	-0.014	0.012	-1.247	0.213	0.875	30.823	0.000	0.011

[1] R.M. Reitan, & D. Wolfson (1985). The Halstead–Reitan neuropsycholgical test battery: Therapy and clinical interpretation. Neuropsychological Press, Tucson, AZ.

[2] Ritchie, K., Roquefeuil, *g.* de, Ritchie, C., *et al.*: COGNITO: computerized assessment of information processing. *J Psychol Psychother.* 2014; 4(2): 1000136.

[3] A. Smith. (1991). Symbol digit modalities test. Western Psychological Services, Los Angeles, CA

[4] https://biobank.ctsu.ox.ac.uk/crystal/crystal/docs/Pairs.pdf

[5] Benton A, Hamsher K, Sivan A: Multilingual aphasia examination. 3rd ed. San Antonio, TX: Psychological Corporation; 1994.

[6] Raven JC: Raven's progressive matrices and vocabulary scales. Oxford, England: Oxford Psychologists Press Ltd. 1989.

[7] Wechsler, D. (1997b) Wechsler Adult Intelligence Scale-Third Edition (WAIS-III). San Antonio: Harcourt Assessment Inc.

[8] Wechsler, D. (1997a) Wechsler Adult Intelligence Scale-Third Edition (WAIS-III). San Antonio: Harcourt Assessment Inc.

[9] Nelson, H.E. & Wilson, J. (1991). National Adult Reading Test (NART), Windsor, UK.

[10] Wechsler, D. (2001). Wechsler test of adult Reading. Psychological Corporation, San Antonio, TX.

[11] Lezak, M.D., Howieson, D.B., Loring, D.W., Hannay, H.J. & Fischer, J.S. (2004) Neuropsychological assessment (4th ed.), Oxford University Press, New York, NY.

[12] Smith, A. (1991). Symbol digit modalities test. Western Psychological Services, Los Angeles, CA

[13] Deary, I. J., Simonotto, E., Meyer, M., Marshall, A., Marshall, I., Goddard, N., & Wardlaw, J. M. (2004). The functional anatomy of inspection time: an event-related fMRI study. *NeuroIm*age, *22*(4), 1466–1479. https://doi.org/10.1016/j.neuroimage.2004.03.047

[14] Deary, I. J., Der, G., & Ford, *g.* (2001). Reaction times and intelligence differences: A population-based cohort study. *Intelligence*, 29 (5), 389-399. https://doi.org/10.1016/S0160-2896(01)00062-9
